# Read count-based method for high-throughput allelic genotyping of transposable elements and structural variants

**DOI:** 10.1186/s12864-015-1700-4

**Published:** 2015-07-08

**Authors:** Alexandre Kuhn, Yao Min Ong, Stephen R. Quake, William F. Burkholder

**Affiliations:** Microfluidics Systems Biology Lab, Institute of Molecular and Cell Biology, Agency for Science, Technology and Research (A*STAR), Proteos Building, Room #03-04, 61 Biopolis Drive, Singapore, 138673 Singapore; Depts. of Bioengineering and Applied Physics and Howard Hughes Medical Institute, Stanford University, Clark Center, Room E300, 318 Campus Drive, Stanford, CA 94305 USA; Visiting Investigator, Institute of Molecular and Cell Biology, A*STAR, Singapore, 138673 Singapore

**Keywords:** Genotyping, Transposable element, Structural variation, Next-generation sequencing, LINE-1, Alu

## Abstract

**Background:**

Like other structural variants, transposable element insertions can be highly polymorphic across individuals. Their functional impact, however, remains poorly understood. Current genome-wide approaches for genotyping insertion-site polymorphisms based on targeted or whole-genome sequencing remain very expensive and can lack accuracy, hence new large-scale genotyping methods are needed.

**Results:**

We describe a high-throughput method for genotyping transposable element insertions and other types of structural variants that can be assayed by breakpoint PCR. The method relies on next-generation sequencing of multiplex, site-specific PCR amplification products and read count-based genotype calls. We show that this method is flexible, efficient (it does not require rounds of optimization), cost-effective and highly accurate.

**Conclusions:**

This method can benefit a wide range of applications from the routine genotyping of animal and plant populations to the functional study of structural variants in humans.

**Electronic supplementary material:**

The online version of this article (doi:10.1186/s12864-015-1700-4) contains supplementary material, which is available to authorized users.

## Background

Transposable elements (TEs) represent a variable but often sizeable fraction of genomes (e.g. > 40 % in human [1] and mouse [2], 10 % in drosophila [3], 85 % in maize [4]) and critically shape their organization and function. Most genomes studied to date contain TE families that are currently active. For instance in humans, novel Alu and LINE-1 (L1) retrotransposon insertions can disrupt gene activity and cause genetic diseases [5]. In mice, IAP retrotransposon insertions have been shown to account for over 10 % of spontaneous mutations [6]. This ongoing activity results in high levels of insertional polymorphism, even between individuals of the same population.

Co-option of specific TE functions by host genomes has led to several critical evolutionary innovations like adaptive immunity in vertebrates [7] and placentation in mammals [8]. However, the general functional impact of novel TE insertions remains unclear. For instance, views on novel retrotransposon insertions in humans range from considering them as essentially evolutionary neutral as long as they do not target exons [9] to being important driving forces behind the evolution of new gene regulatory networks [10]. In support of the latter view, functional molecular studies have established that various active TE families contain regulatory elements that affect transcription at neighboring genes or even beyond (for instance by promoting heterochromatin spreading, see e.g. [11]).

Over the last decade, the availability of whole genome sequences and the development of next-generation sequencing methods have yielded large catalogs of specific TE elements and have started to shed new light onto TEs [12]. Surveying TE elements genome-wide and in larger populations is providing novel insights into their functional impact and evolutionary dynamics. For instance many TEs show considerable stratification across populations [13] and some have notable haplotypic structures compatible with recent, positive selection [14]. Larger-scale TE genotyping in more diverse population will provide a better understanding of their population genetics. Large-scale TE genotyping would also allow for association studies of TE insertions with molecular (e.g. transcription, methylation) or organismal phenotypes which, in turn, would help us to understand their functional effects. The recent discovery of retrotransposition in human brain [15] and tumors [16] has also spawned numerous novel questions about retrotransposon biology beyond inherited germ line insertions. Efficient genotyping methods will thus yield further insights into somatic retrotransposition. Finally, from a more applied perspective, TEs provide powerful genetic markers because of their abundance and dispersion across the whole genome. Affordable and high throughput genotyping methods would be useful for the characterization of diversity in natural and selected populations as well as for marker-assisted selection in plant and animal breeding programs [17].

Historically, genotyping of a specific TE has proceeded by site-specific PCR amplification across the insertion site or across the TE-genome boundary (e.g. [18]). Although it is cheap, this method is not convenient for high-throughput analysis when PCR products are resolved using gel electrophoresis. On the other end of the spectrum, genome resequencing can survey a large fraction of TE insertions genome-wide [13]. It has proven to be useful for TE discovery but, paradoxically, has comparatively poor genotyping accuracy [14, 19]. It also remains expensive and therefore it is generally not applicable to the survey of many samples. Building upon previous methods (e.g. transposon display [20]), several targeted sequencing methods have been developed over the last years (e.g. [21, 22]). They have been instrumental in revealing the extent of TE insertions and polymorphisms in humans [12]. These methods amplify TE junctions by genome fragmentation, adapter ligation and PCR amplification, or by direct amplification using hemi-specific PCR. With regard to genotyping, they are more accurate than whole-genome sequencing [14, 19]. However, owing to the nature of the enrichment scheme, they are restricted to the amplification of a specific TE family. Also, they might be blind to specific insertions with particular flanking sequence properties because they rely on specific sets of degenerate primers to amplify the TE-genome junction (in conjunction with a TE-specific primer) or use enzymatic digestion to create fragments containing TE-genome junctions. Moreover, targeted resequencing necessitates considerable sequencing depth in order to reliably detect the presence of a specific TE insertion. Finally, TE genotyping could rely on SNPs around TEs and presenting high linkage disequilibrium with the insertion allele so that they can be used as proxies to detect TE insertions. Based on this scheme, genotyping could be conducted in a high-throughput manner at very reasonable price because of the availability of SNP array (and other SNP genotyping) technologies. Focusing on L1 elements of the L1Hs family in humans, we found that tagging SNPs likely exist for a majority of insertions [14]. However, we also observed that L1Hs elements could not be systematically assayed by a standard SNP array as it only comprised a minority of L1-tagging SNPs [14]. This is likely to be the case for other important TE families as TEs are usually masked during identification and selection of tagging SNPs.

Here we demonstrate a highly accurate, automated and high-throughput TE genotyping method and apply it to the genotyping of L1 and Alu insertions in humans. It relies on high-throughput sequencing of multiplex, site-specific PCR amplification of TEs of interest. Sequencing reads obtained from PCR products are used to ensure product specificity and make reliable, read count-based, genotype calls. Sequence information around the insertion site yields allelic information and can be used for haplotype analysis. The method is flexible, does not require lengthy optimization rounds and should be within reach of many laboratories owing to the growing availability and affordable costs of high-throughput sequencing. In principle, it can be used to genotype any structural variant that can be assayed by breakpoint PCR amplification [23, 24].

## Results

### Principles of read count-based genotyping

**Fig. 1:**
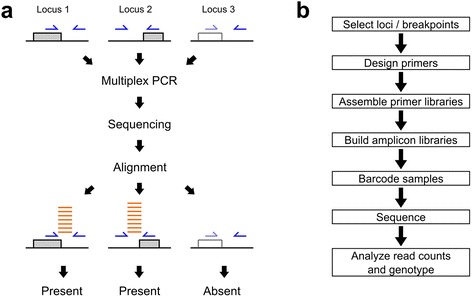
Principle of count-based genotyping of structural variation (**a**) and workflow of a genotyping experiment (**b**). a: Genotyping of three polymorphic TE insertion loci. Gray and dotted boxes represent present and absent insertions, respectively. Blue arrows represent PCR primers and orange lines depict sequencing reads

We describe the principle of the method as applied to the genotyping of TE insertions (Fig. 1a). A set of TE loci is assayed by multiplex PCR amplifications targeting the junctions between TEs and their flanking genomic insertion sites. High multiplexing can be achieved by various (possibly combined) methods including the careful design of primers that allow for multiple PCR reactions in the same reagent volume and parallelization of PCR reactions using droplet or microfluidic technology. Amplicon libraries are sequenced, and reads matching the targeted locations are counted: a high number of specific reads indicates that the corresponding PCR reaction amplified the targeted TE junction and that the TE was present whereas the absence of specific reads (or the presence of a small number of cross-contaminating reads) indicates that the TE was absent.

The workflow of a read count-based genotyping experiment (Fig. 1b) starts with the selection of loci of interest and the design of appropriate primers for each locus. Several separate multiplex PCR libraries are then assembled so that primers used in the same reaction do not form strong dimers that would prevent homogenous amplification of the individual PCR reactions. Here, we typically assembled libraries of 10 to 50 PCR reactions and used a commercial microfluidic chip to build up to 48 10-plex to 50-plex libraries in parallel. The libraries obtained for each sample are then combined and subjected to a second round PCR that adds a barcode and the end adapter sequences required for high-throughput sequencing. Sample-specific libraries are sequenced and demultiplexed. Finally, the specific read count statistics are obtained by bioinformatics analysis and form the basis for making genotype calls.

### Automated allelic genotyping of L1 insertions

**Fig. 2:**
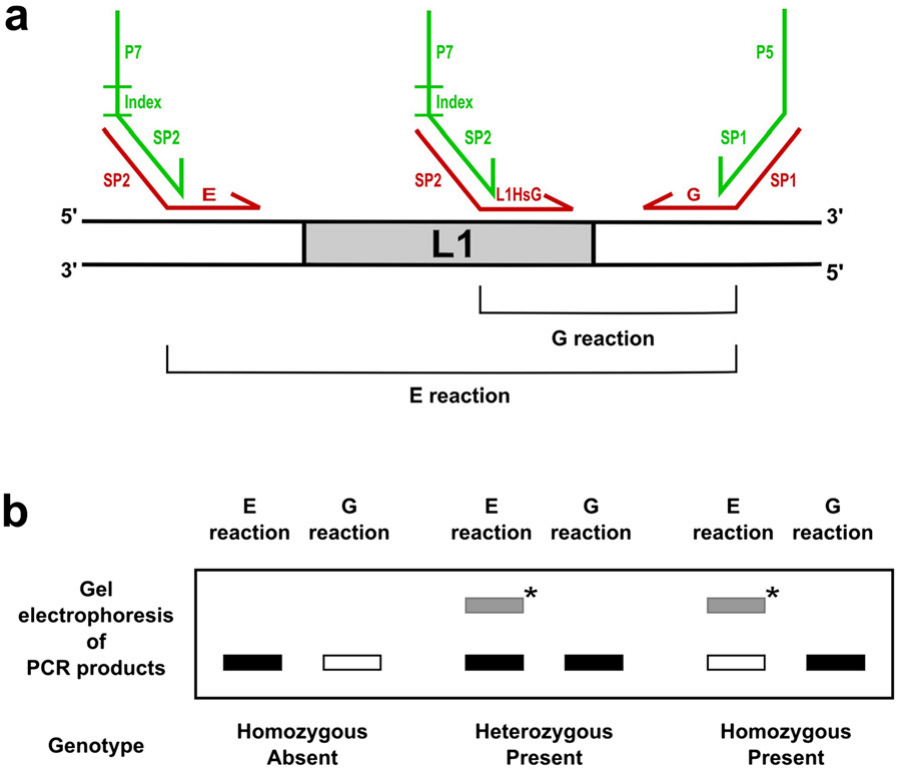
Primers and PCR reactions for allelic genotyping of an L1 element (**a**) and standard gel electrophoresis analysis of the E and G reaction products (**b**). a: The E reaction uses primers located in each of the L1 flanks and tests for the presence of an allele without the L1 insertion. The G reaction uses an L1-specific primer and the primer in the 3’ flank and tests for the presence of an allele bearing the L1 insertion. Primers used in the first PCR (red) are tailed (SP1 and SP2 sequences) so that the adapter (P5 and P7) and index sequences can be added in a second round PCR (green primers). b: Three possible diallelic genotypes based on the presence or absence of the E and G reaction products on an electrophoresis gel. Black filled and empty boxes represent respectively, the presence and absence of a PCR product. The gray filled boxes (marked with an asterisk) represent the (longer) product from the E reaction that is generated in the presence of an allele bearing a short L1 insertion

We first applied our method to the genotyping of L1 insertions. In order to distinguish individuals with homozygous insertion alleles (“homozygous present”) from heterozygous individuals, we adapted the standard scheme requiring two PCR reactions per insertion locus (Fig. 2a). We combined this binary presence/absence read-out scheme with our read count-based method to reliably and efficiently scale up the analysis over many sites and samples. The “E” (for empty) reaction uses site-specific primers in the flanks on each side of the insertion and the “G” (for genomic) reaction uses an L1-specific primer at the 3’end of the element and the site-specific primer in the 3’ genomic flank of the insertion (Fig. 2a). On an electrophoresis gel, an allele that does not carry the L1 insertion yields a product for the E reaction and no product for the G reaction whereas an allele bearing the L1 insertion yields a product for the G reaction but generally no product for the E reaction because L1 insertions are long and prevent efficient amplification. Together the two reactions can thus differentiate the three possible diallelic genotypes (Fig. 2b). The primers for the E and G reactions are tailed with universal sequences (SP1 and SP2) to allow for the second round PCR that adds barcodes (uniquely identifying each individual) and high-throughput sequencing adapters (Fig. 2a).

We tested the method by genotyping 60 human-specific L1 insertions. We designed primer pairs flanking each targeted L1 insertion that we used in conjunction with a single L1-specific primer. The single L1-specific primer was identical across all L1 insertions since all targeted L1 insertions are of the same family (L1Hs) and have an identical 3’ end sequence. We then assembled 12 libraries (6 E and 6 G libraries) targeting 10 L1 insertions each (Additional file 1: Table S1) and assayed 12 samples in parallel using a Fluidigm Access Array chip. We then used a second PCR to barcode the pooled products of the 12 libraries obtained for each sample (Fig. 2a and Additional file 2: Figure S1a) and sequenced the 12 samples in paired-end mode on a MiSeq Illumina benchtop sequencer. Read 1 proceeded from the primer in the 3’ L1 flank whereas read 2 proceeded from the primer in the 5’ flank (E reaction) or the L1-specific primer (G reaction). For each demultiplexed sample, we computationally sorted reads based on their locus of origin and reaction type (E or G) by matching the start of read 1 to each of the 60 3’ flank primers and the start of read 2 to each of the 60 5’ flank primers (E reactions) or the L1-specific primer (G reactions) (Additional file 3: Figure S2). We then discarded reads that did not align to their targeted loci which allowed us to avoid counting reads that arose from unspecific amplification products.

Note that L1 insertions are sometimes 5’ truncated and can be very short. In that case, the E reaction will yield a product despite the presence of the intervening insertion (Fig. 2b). When performing genotyping by gel electrophoresis, knowledge of the product size expected in the absence of the insertion is required to make a correct call and avoid misinterpreting the presence of a product as absence of the insertion. For read count-based genotyping, we introduced the following check: we identified reads spanning small insertions (and giving rise to spurious, high read counts) by detecting L1-specific sequence in the sequencing reads arising from the E reaction and discarding them. L1 elements that required this additional check in order to be correctly genotyped typically were less than 200 bp long.

**Fig. 3:**
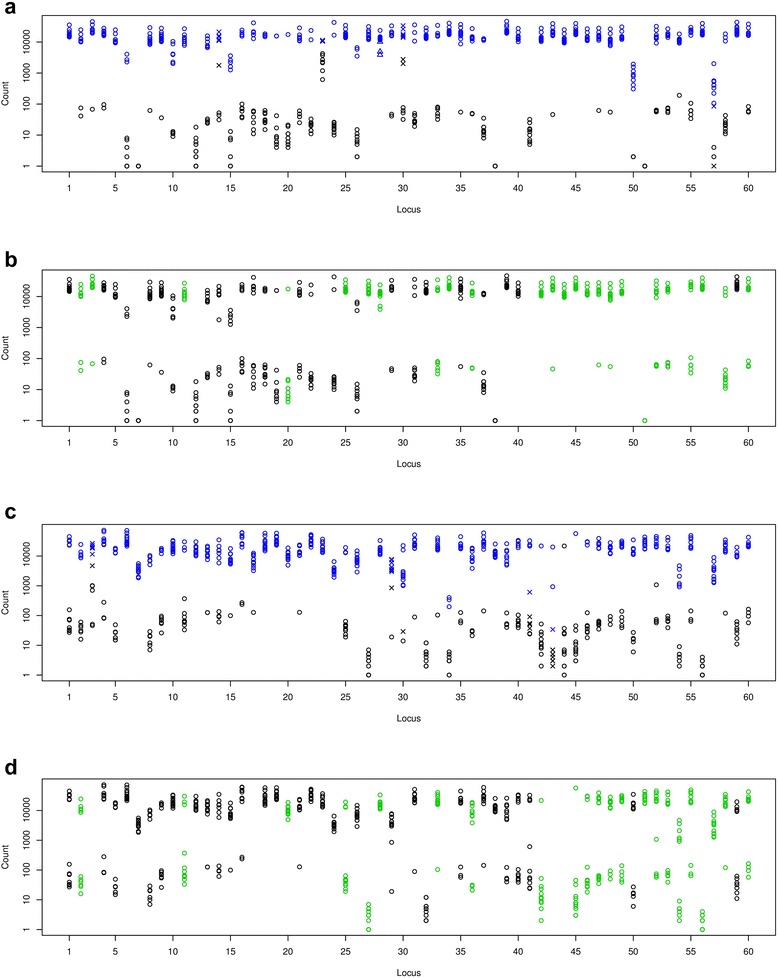
Read counts, automatic genotype calls and validation results for the 60-loci libraries. **a**: Specific read counts for E reactions for 12 samples at each of 60 L1 loci. Blue and black circles represent, respectively, the present and absent calls made based on the clustering of read counts. Crosses indicate genotypes with a quality score less than 7. Triangles (locus 28) indicate genotypes that would be called “present” (blue) because of high read count but that were called “absent” because the L1 sequence was detected in the reads (in the case of very short L1 insertions). **b**: Specific read counts obtained for E reactions for loci that passed quality control. Green and red circles indicate, respectively, concordant and discordant calls for 25 loci that were validated individually using single-locus PCR reactions and gel electrophoresis. All calls were concordant. Locus 41 was excluded from the analysis because the primers did not work. **c**: Same as a but for the G libraries. **d**: Same as b for 23 loci that passed quality control and that were individually validated. All calls were concordant

The specific read counts obtained for E reactions at each locus clearly clustered into two groups (Fig. 3a). The high and low read count clusters comprised, respectively, samples in which the targeted L1 insertion was absent on at least one allele (high), or present on both alleles (low). For most loci, the separation was more than 2 log10 units. G reactions also yielded well separated clusters (Fig. 3c). Here, the high and low read count clusters comprised, respectively, samples in which the L1-bearing allele was present at least once (high) or was absent (low). The exact position of both clusters varied from locus to locus, owing to systematic differences in PCR amplification efficiency. We implemented a locus-specific, unsupervised clustering method to obtain automatic genotype calls (blue and black symbols in Fig. 3a, c).

### Quality controls, validations and genotyping accuracy

**Fig. 4:**
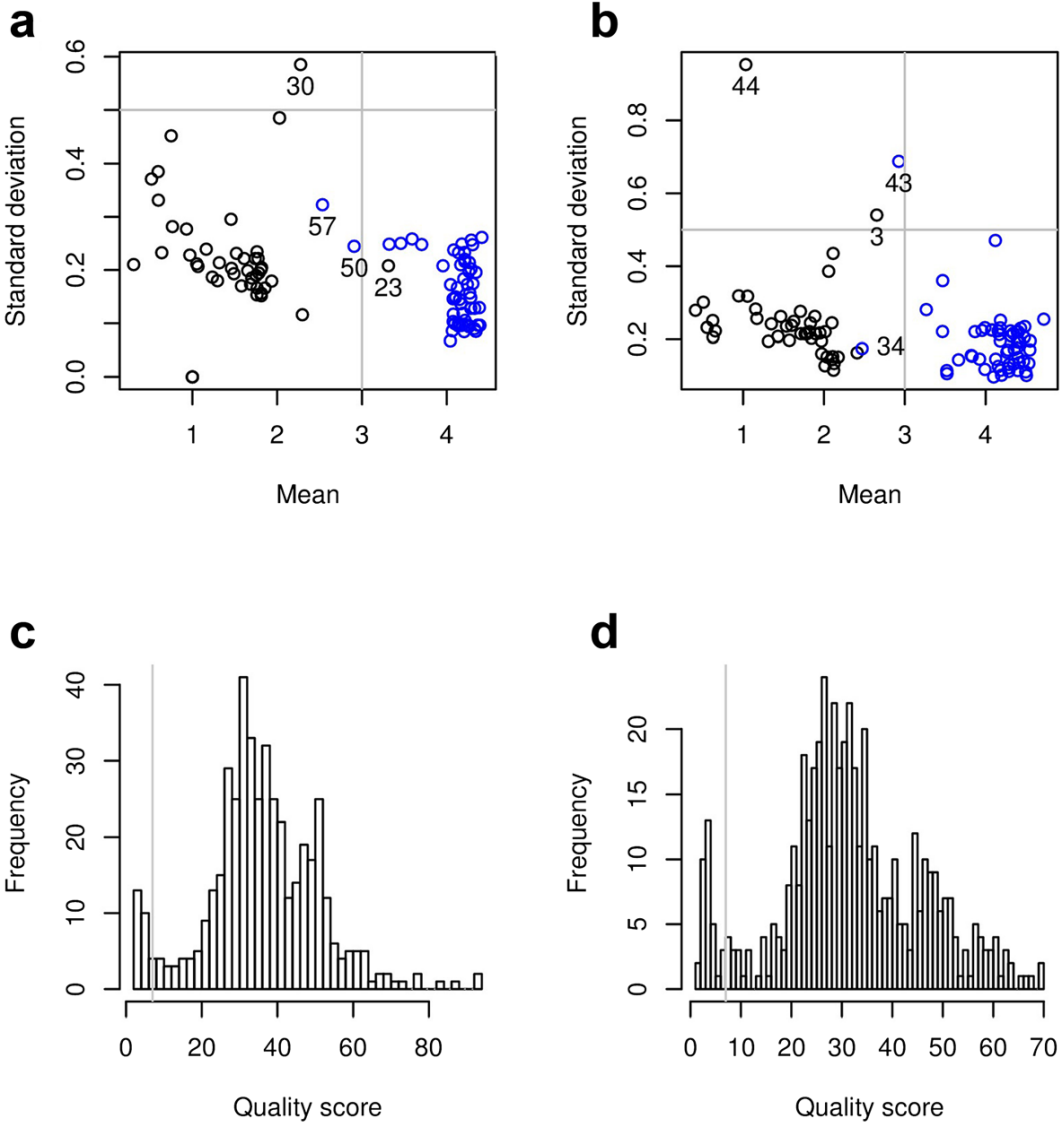
Read count cluster statistics and genotype quality scores for the 60-loci libraries. **a**: Mean versus standard deviation of clusters obtained with the E libraries. Black and blue circles indicate, respectively, low and high read count clusters. Despite locus-to-locus variations, most clusters had similar means and standard deviations. We manually set thresholds (represented as gray lines) at 3 (mean) and 0.5 (standard deviation), which dropped out locus 23 (low read count cluster had mean greater than 3), loci 50 and 57 (high read count cluster had mean less than 3) and locus 30 (standard deviation greater than 0.5). **b**: Same as a for the G libraries. We dropped locus 34 (high read count cluster had mean less than 3) and loci 3, 43 and 44 (standard deviation greater than 0.5). **c**: Histograms of genotype quality scores obtained for the E libraries. Scores below 7 (threshold indicated by a gray vertical line) are indicated as crosses in Fig. 3a, c. **d**: Same as c for the G libraries

We used the position and spread of the low and high read count clusters to automatically spot loci with potential genotyping problems (Fig. 4a-b). Unusual cluster mean (excessively low mean for a high read count cluster or excessively high mean for a low read count cluster) signaled loci that did not amplify convincingly (e.g. locus 50 in Fig. 3a) or that failed clustering (e.g. locus 23 in Fig. 3a). We dropped 6 and 7 loci from the E and G libraries, respectively: For the E libraries, 4 loci showed poor clustering (Fig. 4a) and 1 locus had reads that did not map uniquely to the targeted site. For the G libraries, 4 loci showed poor clustering (Fig. 4b) and 2 loci had reads that did not map uniquely. One primer pair was found a posteriori not to work properly and was excluded from both libraries. In addition to characterizing each locus using statistical characteristics of the clusters, we also derived genotyping quality scores representing the confidence of each call given the observed read count and the underlying clusters (Fig. 4c-d). Loci where many samples showed low quality scores overlapped with loci dropped based on poor cluster characteristics (Fig. 3a, c).

We obtained allelic genotype calls for loci for which both the E and G reactions passed quality checks (Additional file 4: Table S2). We had previously performed individual PCR and gel electrophoresis for 29 of these loci [14]. We thus compared the read count-based genotypes obtained here with the genotypes calls obtained with the standard gel-based method: For the E libraries, all 300 tested genotype calls (25 previously genotyped loci that passed quality control times 12 samples) were concordant (Fig. 3b). For the G libraries, all 276 tested genotype calls (23 previously genotyped loci that passed quality control times 12 samples) were concordant (Fig. 3d).

Further, we aimed to assess the accuracy of our read-count based genotyping method on an independent dataset and set out to genotype 22 additional L1 insertions across 24 samples (Additional file 1: Table S1, Additional file 2: Figure S1b, Additional file 5: Figure S3). 18 and 17 loci passed quality control for the E and G libraries, respectively (Additional file 6: Figure S4). To compare with the read count-based genotypes (Additional file 7: Table S3), we performed individual PCR reactions and gel electrophoresis analysis for all 22 loci (Additional file 8: Figure S5). We found 6 genotyping errors across the E and G libraries. All but one were found at locus 19 (“P1_M_061510_20_89”). The four samples that were miscalled in the E reaction of locus 19 actually had a high read count but the corresponding tags did not perfectly align to the target location so that their specific read count was low. Read tags are extracted from the beginning of reads and are used to ensure specificity of amplification products (see Methods). Upon realignment of the tags to the genome, we found that the problem was caused by a known 1-bp deletion variant (rs55989974) in these four samples specifically (NA07037, NA07051, NA11830, NA11992). Allowing for slight misalignments of the tags to the target location hence resulted in a correct genotype call in this case. We also examined the error at locus 16 (E libraries) and found that it was caused by an error in primer design. The 3’-flank primer overlapped a known SNP which prevented the efficient amplification of the E reaction on the allele that did not carry the L1 insertion (Additional file 8: Figure S5, “P1_M_061510_14_175”, page 20).

Thus the concordance between read-count based genotypes and genotypes obtained from standard, individual PCRs was 99.8 % for both the E and G libraries (out of 432 genotype calls, i.e. 18 loci times 24 samples for the E libraries and 408 genotype calls, i.e. 17 loci times 24 samples for the G libraries). These results demonstrate the very high accuracy of read count-based genotyping.

### Automated genotyping of Alu insertions demonstrates assay scalability

We aimed to assess the flexibility and scalability of our method and set out to genotype another type of polymorphic transposable element. We focused on the currently active AluYb8 subfamily of Alu elements. Alu insertions play an important role in genome biology as they create genome diversity, influence gene expression and can generate disease-causing mutations [25].

We asked if PCR reactions performed in the Access Array could sustain higher levels of PCR multiplexing. We designed primers and assembled them in 47- and 57-plex reactions, assaying a total of 104 Alu insertion loci across 40 DNA samples (Additional file 1: Table S1). Despite higher multiplexing, sequencing libraries did not show unspecific products when analyzed by gel electrophoresis (Additional file 2: Figure S1). Upon sequencing, the vast majority of loci showed excellent separation between high and low read count clusters for both the E and G libraries (Additional file 9: Figure S6 and Additional file 10: Figure S7), allowing us to make 3,480 high quality genotype calls (Additional file 11: Table S4).

We compared our genotype calls with calls previously obtained by the 1000 Genome Project (1000GP) [13]. Focusing on high-quality calls from the 1000GP (i.e. GQ > 10), we identified 68 (out of 3,158) and 84 (out of 3,360) discordant calls in the E and G libraries, respectively. Upon individual PCR validation, we found that only 16 calls in the E library and 42 calls in the G library were actual genotyping errors in our experiment (which also suggests that genotyping accuracy for Alu insertions in the 1000GP was much better than overall accuracy aggregated over several transposable elements and, in particular better than for L1s). This confirms the very high accuracy (>99 %) of our genotyping method (Additional file 12: Figure S8). Finally, only 16 loci (15 %) and 8 loci (8 %) were eliminated upon quality check of the E and G libraries, respectively and the drop-out rate for combined (allelic) calls was 16 %. The reasons for dropping loci were either lower separation between read count clusters or read alignment to multiple locations in the genome. However, when compared to individual PCR validations, most of the eliminated loci showed error-free genotype calls (not shown), indicating that our criteria for quality assessment were quite strict and that the drop-out rate could be decreased further.

In conclusion, high accuracy levels and low drop-out rates were maintained at higher levels of PCR multiplexing. Importantly, we designed primers automatically and assembled them in multiplex reactions without recursive optimization rounds, highlighting the efficiency of the method. As we used 4 out of 48 channels of the Access array to build the 4 libraries (2 paired E/G libraries), these results show that the method can scale up to over 1,000 loci (50 loci × 24 channels) across 48 samples on a single Access Array chip.

### Phasing and haplotype analysis of L1 insertions

In addition to accurate genotyping, many potentially powerful applications require the phase of variants to be known, i.e. to determine which of the two alleles at a heterozygous locus bears the variant, such as TE insertion in the case of L1 and Alu genotyping. This is critical for analyzing the haplotypic structure around structural variants. The sequence immediately flanking the 3’ end of the insertion that is captured by the PCR reactions and is contained in sequencing read 1 can be used to determine the phase of insertions. Specifically, we looked for known SNPs in this sequence (located between the end of the 3’ flank primer and the 3’ end of the insertion) and for each sample, we called heterozygous SNPs in the sequencing reads from the E and G reactions separately. We then compared these calls to the known SNP alleles to identify the insertion-bearing allele.

The HapMap samples used in our experiments were included in the 1000 Genome project (1000GP) and we set out to phase heterozygous samples in our validation (22-loci) experiment with the SNP data provided for these samples by the 1000GP. The length of the sequence between the end of the 3’ flank primer and the L1 3’ end varied between 274 and 350 bp (mean 310 bp) across the 22 loci and contained from 1 to 11 SNPs (mean 5.2) as identified and genotyped by the 1000GP project. Mining the SNP calls provided by the 1000GP, we found that one third (47) of all heterozygous L1 samples (129) had at least one heterozygous SNP in the accessible L1 flanking sequence and could thus potentially be phased. Upon SNP calling in the E and G reactions separately, all of the heterozygous L1 samples with at least one heterozygous SNPs could be successfully phased: the SNP alleles called in the E and G reactions always matched the two alleles called by the 1000GP. Moreover, in the case of more than one heterozygous SNP in the flanking sequence, each SNP resulted in the same phase call, demonstrating the reliability of the procedure.

**Fig. 5:**
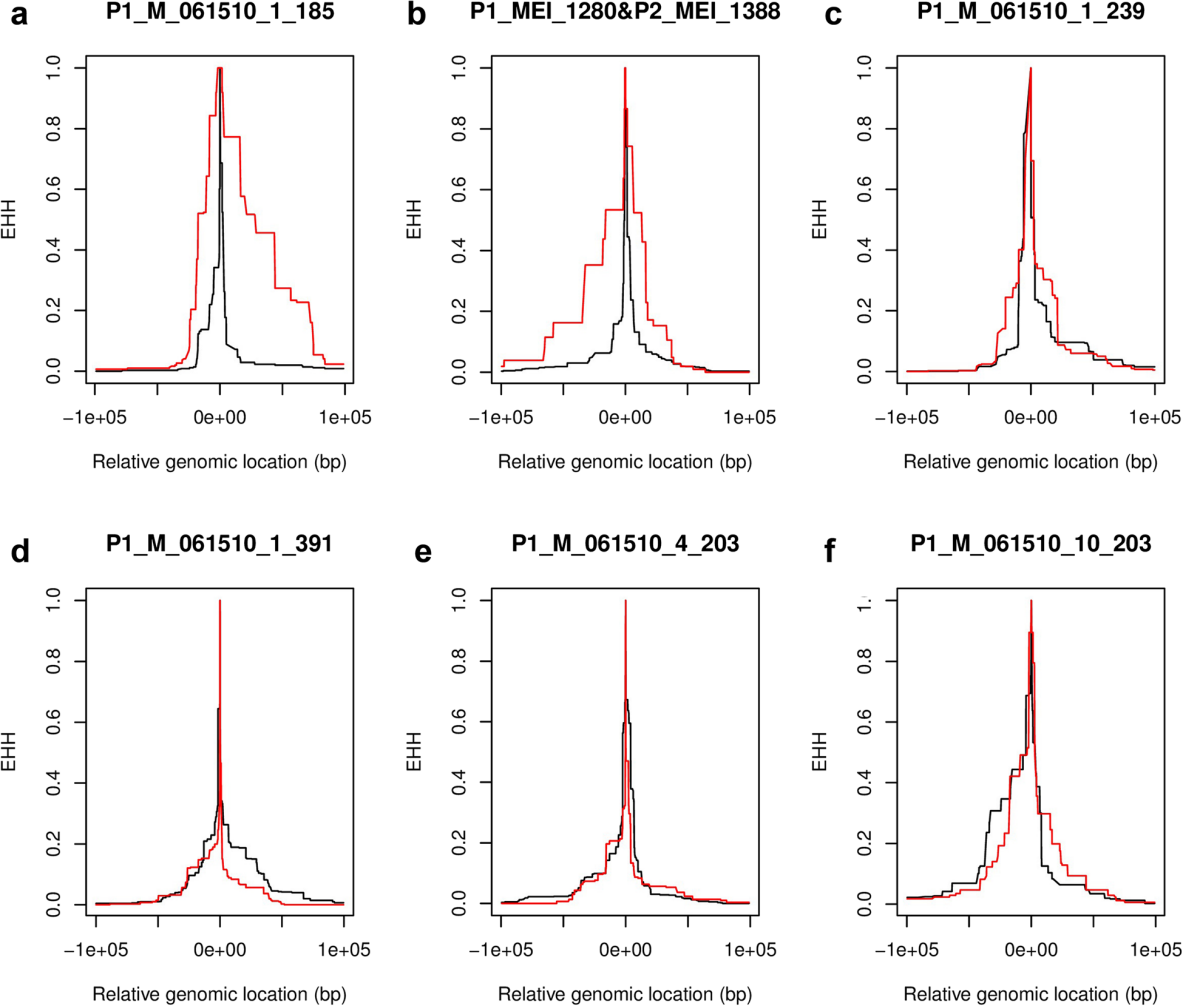
EHH analysis in the 100 kb regions around 6 selected L1 insertions (included in the 22-loci libraries) using the genotypes and phase information obtained from 40 CEU samples. P1_M_061510_1_185 (**a**) and P1_MEI_1280&P2_MEI_1388 (**b**) show clear haplotype differences between the L1-bearing allele (red) and the allele without insertion (black). The remaining insertions (**c-f**, corresponding to P1_M_061510_1_239, P1_M_061510_1_391, P1_M_061510_4_203, P1_M_061510_10_203, respectively) do not show clear haplotype differences between both alleles

We used the phase information obtained here to analyze the haplotypic structure around a small set of L1 insertions. Haplotypic structures can provide useful information on the evolutionary dynamics of genetic variants: We previously detected significant extended haplotype homozygosity (EHH) around particular L1 insertions, compatible with the signature left by recent and rapid positive selection events. To ensure a high genotyping accuracy, we had genotyped these L1s using individual, site-specific PCRs and gel electrophoresis analysis. Because this standard genotyping approach cannot yield L1 phases, however, we had to restrict our analysis to homozygous samples only. Here, we repeated the EHH analysis around 7 L1 insertions that we included in the 22-loci experiment and that had been previously analyzed. We used L1 genotypes and phase information obtained with our sequencing-based method, resulting in the additional inclusion of phased heterozygous samples in the analysis. We included the 24 samples assayed for the validation experiment and performed read count-based genotyping on 16 additional samples to obtain a cohort of 40 samples. For each locus, the phase information allowed us to include between 1 and 15 additional (heterozygous) samples in the analysis (depending on the number of heterozygous L1 samples containing at least one heterozygous SNP in the L1 flanking region). Out of the 7 L1 insertions assayed here, 6 were successfully genotyped and phased and two showed strong differences in EHH signals obtained for the alleles with (red) and without (black) L1 insertion, reflecting haplotypic differences on both alleles (Fig. 5). They indeed correspond to 2 of the 3 L1s identified in our previous study (the third L1 insertion was not included in the 22-loci libraries and hence could not be analyzed) and that might have been under positive selection in recent human evolution.

## Discussion

We have presented a large-scale genotyping method for insertions and other types of genetic variants that can be assayed by PCR. The information yielded by standard electrophoresis analysis of PCR reactions is restricted to the presence and size of one (or a small number of) product(s). Instead, we used high-throughput sequencing as a PCR read-out method. We showed that the sequence information and quantitative read counts obtained by sequencing allows for highly accurate, robust, flexible and large-scale genotyping as well as haplotype analysis. When compared to individual PCR reactions resolved on agarose gels, the concordance of the method as applied to L1 genotyping was above 99.8 %. We showed that improved primer design can further increase concordance, in particular taking into account known SNPs when designing primer sequence. For comparison, we and others previously estimated that the accuracy of L1 genotyping based on whole-genome resequencing or targeted resequencing (e.g. L1-seq) was around 90 % [13, 14]. Notably, we have previously shown that this accuracy level can confound genetic analyses (as demonstrated in our study of L1-taggability [14]), highlighting the need for highly accurate genotyping methods.

L1 insertions are long (6 kb) structural variants (albeit they are sometimes truncated). Alu insertions, on the other hand are short (300 bp) elements that are often found in gene regions and sometimes nested in other repetitive elements. They thus represent a challenging and interesting test for our sequencing-based method. We profiled a set of Alu insertions using 50-plex PCR reactions and showed that our method can scale up to over 1,000 loci (per Access array) without increasing drop-out rate or compromising genotyping accuracy. Smaller indels (<100 bp) could be assayed with a single E library as both alleles (with and without the insertion) will amplify similarly and could thus be differentiated via detection of an actual insertion tag (similarly as for the detection of L1 or Alu “tags” for the E genotyping of short, truncated L1/Alu insertions). Importantly, all the primers in our libraries were designed and assembled in a single round, without iterative optimization.

The drop-out rate in read count-based genotyping varied between 10 % (60-loci libraries) and 20 % (22-loci libraries). A common reason for locus exclusion was excessive read count variability (Additional file 6: Figure S4). This was caused by unreliable PCR reactions (e.g. locus 15, Additional file 5: Figure S3a) or failed statistical clustering (e.g. locus 22, Additional file 5: Figure S3c). The former can be addressed experimentally by redesigning primers or by regrouping primers into different libraries, since primer pairs usually (but not always, see below) worked when used in single PCR reactions (see locus 15 “P1_M_061510_13_47” in Additional file 8: Figure S5). Improved approaches for predicting primer interactions will thus be helpful in routinely designing larger multiplex libraries. Cases of failed statistical clustering can be addressed by refining the computational analysis method. Indeed, we observed several loci with an inflated standard deviation caused by a small number of samples with an intermediate read count. Improving the handling of such outliers using refined clustering methods could thus decrease dropout rate without compromising accuracy. Finally, note that the dropout rate for standard genotyping by individual PCR reactions analyzed on gels was smaller compared to read count-based genotyping, yet it was significant: for the 22-loci experiment, for instance, we had to redesign primers for 2 loci, because they produced multiple products that did not allow for unambiguous genotyping by electrophoresis analysis (see “P1_M_061510_8_220” and “P1_M_061510_20_89” in Additional file 8: Figure S5). Importantly, the original primers actually yielded genotypes with the specific read count-based method, highlighting the benefit of using sequence information for genotyping.

The costs of materials and reagents for read count-based genotyping are dominated by sequencing costs (Additional file 13: Table S5). The cost per genotype thus decreases with the number of loci and samples assayed. In contrast, the cost per genotype using standard genotyping on agarose gels is fixed and mostly determined by the costs of DNA polymerase. Based on the use of the Access Array for the preparation of 10-plex libraries, we considered the costs of experiments of various scales. Whereas the cost per genotype was about 5 times higher for read count-based genotyping compared to standard genotyping in small scale experiments, it was more than twice cheaper when making full use of the chip (compare e.g. “12 libraries and 12 samples” and “48 libraries and 48 samples” in Additional file 13: Table S5). Increasing the multiplexing of individual libraries (e.g. from 10-plex to 100-plex PCR) reduces costs proportionally and results in dramatic economies of scale (Additional file 13: Table S5). However, increasing multiplexing usually requires further optimization in library design. The automatic library preparation system (here the Access Array) is the second cost-determining factor of read count-based genotyping and it might seem advantageous to replace it with manual preparation. The very small volumes of enzymatic reagents required in the Access Array (3 μl), however, makes the manual solution cheaper for small scale experiments only (“12 libraries and 12 samples”, Additional file 13: Table S5). Two factors were excluded from the analysis: Primer synthesis costs will be higher for read count-based genotyping, because the required primers are longer and a second-round PCR with barcoding primers is necessary. Second, we did not take into account labor costs. If labor costs were included, the much shorter handling time of automated library preparation compared to standard genotyping on gels could greatly shift the balance in favor of read count-based genotyping. In conclusion, read count-based genotyping can be cost- and time-effective for medium scale experiments already, i.e. targeting a few hundred loci across a few dozen of samples for instance.

Finally, we summarize important practical considerations for library design, preparation and analysis:

1. Amplicon size is not fixed and can be adapted to the target application. In the 60-loci experiment, we used 200 bp amplicons but shorter amplicons can be used. The minimal length is determined by the length of the tag used to verify read specificity (here 50 bp). In the 22-loci experiment, on the other hand, we used 400 bp amplicons to increase the number of sequenced SNPs and thus improve haplotyping.
2. It is critical, however, that amplicons in a particular multiplex library be homogeneous in size because we observed that the presence of shorter amplicons compromised the homogeneous amplification of libraries. We observed that this effect was much stronger than having primers with variable GC content (not shown). As a matter of fact, we allowed for a wide range of GC content (corresponding to melting temperatures of 57 to 65 °C) but strongly constrained amplicon size differences to a maximum of 200 bp (but actual size differences were much less for most amplicons, see Additional file 2: Figure S1).
3. The 2-round PCR scheme is used to add sample-specific barcodes and harness the huge throughput of a sequencing run. It also permits the use of shorter (and hence cheaper) primers because PCR 1 primers then do not have to contain sequencing adapters. This scheme also improves multiplexing, because the second PCR relies on a single pair of sequences (hybridizing to the universal tails of the primers in the first PCR), which was proposed to reduce amplification bias [26].
4. We systematically varied the number of PCR cycles but concluded that fine-tuning is not critical. Indeed, we observed that increasing the number of PCR 1 cycles increased read count variability (not shown), which confirmed the assumption underlying the tailed multiplex PCR method. However, we also observed that increasing the number of PCR 2 cycles increased read counts for absent amplicons (not shown), most probably by amplification of residual contamination. Thus the respective number of cycles for the first and second PCR cannot be set arbitrarily and represents a trade-off between two different sources of read count variability.
5. Independent, experimental validation of primer pairs was necessary for only 2 loci in the 22-loci libraries as they did not show any specific reads for any samples upon sequencing. This could have been due to the absence of the corresponding amplicon in all samples (rare L1 insertion) or to the primers failing to prime the PCR reaction. To rule out the latter, we validated the primer pairs using additional DNA samples and successfully amplified the target from at least one of them, confirming that the primer pair worked as expected (“P1_M_061510_1_131” and “P1_M_061510_18_386” in Additional file 8: Figure S5). Such independent primer validation, however, is mitigated as the number of samples grows larger since the probability of including at least one sample comprising the target (and showing amplification of the targeted product) increases.

## Conclusions

We present an efficient method to perform high-throughput genotyping of TEs and other types of structural variants. We provide a detailed demonstration of how to design, build and analyze sequencing libraries that allow for large-scale genotyping studies. Applying our method to the genotyping of L1 insertions in human samples, we show that it is as accurate as “gold standard” site-specific PCR assays. However, it remainscost-effective and it is cheaper than standard methods for medium and large scale experiments. However, it remainscost-effective and it is cheaper than standard methods for medium and large scale experiments. We demonstrate the flexibility of the method by genotyping a shorter TE (Alu) and show that it can scale up without compromising genotyping accuracy or increasing the drop-out rate. As exemplified by our analysis of the haplotypic structure around L1 insertions, this novel method can benefit a wide range of applications including high-throughput, routine genotyping and functional studies of TEs and structural variants.

## Methods

### Primer and library design

We used Primer3 [27] to design PCR primers flanking L1 insertions (Fig. 2a). Specifically, we retrieved genomic sequences of 100 bp in the 5’ and 3’ flanking regions of each L1 and submitted them to the Primer3 software program to obtain sets of potential 5’- and 3’-flank primers. We used the default Primer3 settings except for the melting temperature (PRIMER_OPT_TM = 61, PRIMER_MIN_TM = 57, PRIMER_MAX_TM = 65). The target sequence for primer design in the 5’ flanking region extended from 110 to 10 bp upstream of the L1. The target sequence for primer design in the 3’ flanking region extended from 70 to 170 bp (60-loci libraries) or from 270 to 370 bp (22-loci libraries) downstream of the L1. For each flanking sequence, all potential primers returned by Primer3 were aligned to the human genome using blat [28] and primers with multiple genomic matches were dropped. We grouped 10 pairs of 5’- and 3’-flank primers (corresponding to 10 L1s) for use in a multiplex PCR reaction (E libraries). The 5’-flank primers of each set were used together with the L1Hs-specific primer in another multiplex PCR reaction (G libraries). We used AutoDimer [29] to check for primer dimers and hairpin interactions among primers of each individual multiplex E library. In the cases where a primer was flagged by the AutoDimer software, the corresponding primer pair was exchanged with a pair from another set, or the primer was replaced by another potential primer chosen by Primer3 or the primer pair was dropped. Finally, 5’- and 3’-flank primers were tailed with the SP2 and SP1 sequences, respectively.

For Alu insertions, we focused on elements of the AluYb8 family that are present in the human reference genome (i.e. the “deletion” set of variants identified and genotyped as part of the 1000 Genome Project [13]) and that had the AluYb8 family-specific 3’ diagnostic sequence ACTGCAGTCCGCAGTCCG. The primer design rules we followed were the same as for the 60-loci L1 libraries. We assembled primer pairs into 2 multiplex groups of 47 and 57 loci (assaying a total of 104 Alu insertions). Primer sequences, genomic locations and primer library composition are shown in Additional file 1: Table S1.

We used L1 and Alu sets that have been previously genotyped by Stewart et al. [13] as the basis set for our study. Their set comprised 82 and 135 high-quality L1s (so-called “deletion” and “insertion” sets in Stewart et al., see also [14]). For the 60-loci libraries, 40 L1s were from the “deletion” set and 20 were from the “insertion” set in Stewart et al. The strand of L1s in the “insertion” set was not determined by Stewart et al. and we could only use L1s for which we had determined the encoding strand ourselves (i.e. for 23 L1s, see our previous study). Thus, all 82 L1s of the “deletion” set were run through our automatic primer design pipeline and we selected the first 40 L1s which successfully passed primer and library design. Similarly, all 23 L1s of the “insertion” set with previously determined strands were run through the primer design pipeline and 20 were used for this study. Regarding AluYb8 insertions, the Stewart et al. study lists a total of 237 of them. Primers were designed for all insertions and we randomly selected 104 Alus that successfully passed primer and library design.

### Library construction and sequencing

To construct multiplexed sequencing libraries on the Fluidigm Access Array, we first prepared a primer mix for each multiplex library such that the final concentration of each primer in the mix was 2 μM (0.5 μM for the 104-loci Alu libraries). Separate primer mixes were prepared for E and G reactions: For the 60-loci libraries we prepared 12 primer mixes corresponding to 6 E and 6 G libraries (probing 10 loci each); for the 22-loci libraries we prepared 6 primer mixes corresponding to 3 E and 3 G libraries (probing 7 to 8 loci each); for the 104-loci Alu libraries we prepared 4 primer mixes corresponding to 2 E and 2 G libraries (probing 47 and 57 loci). 20× Primer Solutions were prepared by adding 5 μl of 20× Access Array Loading Reagent (Fluidigm) to 50μl (95 μl for the 104-loci Alu libraries) of primer mix and topping up with ddH_2_O to 100 μl. Sample Mix Solutions were prepared according to the manufacturer’s instructions using DNA from 12 HapMap samples (NA06986, NA07000, NA07037, NA07051, NA07346, NA07347, NA07357, NA11829, NA11830, NA11831, NA11881, NA11894) for the 60-loci libraries, 24 HapMap samples (NA06986, NA07000, NA07037, NA07051, NA07347, NA07357, NA11829, NA11830, NA11831, NA11894, NA11919, NA11920, NA11931, NA11992, NA11993, NA11994, NA11995, NA12003, NA12006, NA12043, NA12044, NA12045, NA12144, NA12154) for the 22-loci libraries or 40 HapMap samples (NA06986, NA07000, NA07037, NA07051, NA07346, NA07347, NA07357, NA11829, NA11830, NA11831, NA11881, NA11894, NA11918, NA11919, NA11920, NA11931, NA11992, NA11993, NA11994, NA11995, NA12003, NA12006, NA12043, NA12044, NA12045, NA12144, NA12154, NA12155, NA12156, NA12249, NA12287, NA12489, NA12716, NA12749, NA12750, NA12751, NA12761, NA12763, NA12776, NA12828) for the 104-loci Alu libraries. The 60-, 22- and 104-loci libraries were built on separate Access Arrays. In order to fully utilize the 48.48 Access Array IFC, we loaded each Primer Solution and Sample Mix multiple times. For the 60-loci libraries we loaded each of the 12 Primer Solutions into four Primer Inlets and each of the 12 Sample Mix Solutions into four Sample Inlets. For the 22-loci libraries, we loaded each of the 6 Primer Solutions into 8 Primer Inlets and each of the 24 Sample Mix Solutions into two Sample Inlets. For the 104-loci Alu libraries, we loaded each of the 4 Primer Solutions into 12 Primer Inlets. We then loaded the Access Array IFC onto the Fluidigm BioMark HD, ran the standard Access Array protocol and harvested the multiplexed PCR products from the IFC at the end of the run. Each sample harvested consisted of the pooled PCR products from all 120 (60-loci experiment), 44 (22-loci experiment) or 208 (104-loci experiment) individual PCR reactions that used a particular DNA sample as template.

We next diluted each sample 100-fold and used the diluted samples in a second round of PCR which amplified the libraries and incorporated the appropriate Illumina sequencing adapters and barcodes. Each 25 μl reaction consisted of 2× GoTaq Green Master Mix (Promega), 10 pmoles of P5SP1 primer and P7-index-SP2 primer, and 1 μl of the 100-fold diluted samples. The thermal cycling protocol was 95 °C for 5 min, followed by 10 cycles of 95 °C for 30 s, 60 °C for 30 s, 75 °C for 1 min, and ended with 75 °C for 10 min. After the PCR, we ran the products on a 2 % TAE agarose gel and excised fragments between 200 and 1000 bp (60-loci), 400 and 600 bp (22-loci) or 250 and 400 bp (104-loci) which we then gel-extracted and purified using the QIAquick Gel Purification Kit (Qiagen) and eluted in a volume of 50 μl of water. We removed adenine overhangs by incubating the libraries with 6.25U of Pfu Turbo DNA polymerase (Agilent) and 10 nmoles each of dNTPs for 1 h at 72°C. We purified the blunt-ended fragments again with the MinElute Reaction Cleanup Kit (Qiagen) and eluted in a volume of 10 μl of water before analyzing the libraries using the High Sensitivity Chip on the Agilent 2100 BioAnalyzer to determine DNA concentration and quality. The 12 (60-loci) or 24 (22-loci) libraries were pooled in equimolar amounts according to the BioAnalyzer results and subjected to quantitative PCR using the KAPA Library Quantitation Kit (KAPA Biosystems). The 40 libraries corresponding to the 104-loci experiment were quantified individually using the KAPA qPCR kit and were pooled in equimolar amounts according to the qPCR results (which led to more homogeneous representation of each sample in the final sequencing library). We sequenced libraries on the Illumina MiSeq using the MiSeq Reagent Kit v2. For the 60-loci and 104-loci libraries, we used a 300 cycle-kit and ran the pair-end sequencing protocol with 250 cycles for Read 1, 69 cycles for Read 2 and 6 cycles for the Index read. For the 22-loci libraries we used a 500 cycle-kit and ran the pair-end sequencing protocol with 450 cycles for Read 1, 69 cycles for Read 2 and 6 cycles for the Index read.

To increase the number of samples available for the haplotype analysis, we used the 22-loci libraries to assay 16 additional HapMap samples (NA07346, NA11881, NA11918, NA12155, NA12156, NA12249, NA12287, NA12489, NA12716, NA12749, NA12750, NA12751, NA12761, NA12763, NA12776, NA12828) manually. In place of the Access Array, we assembled each of the 6 PCR reactions (3 E and 3 G) for the 16 samples in individual wells of a 96-well PCR plate. We used the FastStart High Fidelity PCR System, dNTPack (Roche), which was also used in the Access Array, to assemble the reactions. Each 25 μl reaction consisted of 10× FastStart High Fidelity Reaction Buffer without MgCl_2_, 112.5 nmoles of MgCl_2_, 5 % DMSO, 5 nmoles each of dNTPs, 2.5 μl of Primer Mix (2 μM each primer) and 100 ng of DNA. We used the same thermal cycling protocol as the Access Array for the amplification. After thermal cycling, we pooled the 6 reactions obtained for each sample and diluted them 100-fold. We then proceeded with the second round of PCR as we did for the Access Array protocol. The 16 libraries were sequenced in a separate MiSeq sequencing run.

### Processing of sequencing data and read counts

Samples were automatically demultiplexed by the MiSeq sequencer. We sorted reads from each sample according to the targeted E and G amplicons (Additional file 2: Figure S2). Specifically, we identified reads arising from a given targeted G amplicon as (paired-end) reads with a perfect match to the 3’-flank primer at the beginning of read 1 and a match (with maximal mismatch of 1 bp) to the L1Hs (L1Hs-specific primer, 60- and 22-loci libraries) or AluYb8 (AluYb8-specific primer, 104-loci libraries) sequence at the beginning of read 2. We identified reads arising from a given targeted E amplicon as (paired-end) reads with a perfect match to the corresponding 3’-flank primer at the beginning of read 1 and a perfect match to the corresponding 5’-flank primer at the beginning of read 2. For each set of reads identified as originating from a particular amplicon, we then extracted sequence tags defined as 50 bp-sequences immediately downstream of the 5’-flank primer sequence in the read. We used these tags to check for amplicon specificity. We aligned sequence tags using bowtie2 [30] and recorded the number of aligned and uniquely aligned tags to assess mapping success and specificity, respectively. We then compared the position of aligned tags to the location of the targeted amplicon. We recorded the number of tags aligning exactly to the targeted genomic location and used it to call the presence of the targeted amplicon and generate genotype calls.

In the (rare) cases of very short (< 200 bp), truncated L1 or Alu elements, E amplicons could show high numbers of specific reads despite the presence of the L1 element because the amplicon spanning the insertion was not much longer than the amplicon produced in the absence of the insertion. To detect and correct for such cases (i.e. to avoid making an L1 or Alu absence call in this case), we counted for each E amplicon the number of reads containing an L1 or Alu sequence. Specifically, we classified reads as containing an L1 or Alu element if there was a match to, respectively, the reverse complement of the L1Hs-specific sequence TGCACATGTACCCTAAAACTTAG or the reverse complement of the AluYb8-specific sequence ACTGCAGTCCGCAGTCCG (allowing for a maximum of 6 mismatches). A high number of reads (> 80 % of all reads) with an L1 or Alu sequence was used to diagnose L1 or Alu presence (and change the call from “absent” to “present”, see below). Unless indicated, analyses were performed in R/Bioconductor [31, 32], including packages ShortRead [33], rtracklayer [34] and VariantAnnotation [35].

### Automatic genotype calls, quality control and quality scores

The presence of a particular amplicon was associated with a large number of specific reads (as assessed by the genomic alignment of read tags) whereas its absence was associated with a lower number (or total absence) of specific reads. The number of reads associated with the presence of a given amplicon could vary across different amplicons and/or experiments. Read counts were influenced by many factors including primer efficiency, number of PCR cycles and sequencing throughput. When multiple samples were considered simultaneously, however, specific read counts clustered around a high and a low count level, corresponding respectively to the presence and the absence of the amplicon. For each amplicon, we used a Gaussian mixture to model (log 10) specific read count measured for each sample and automatically classify each sample into the high (present) or low (absent) count cluster. Read counts of 0 were set to 1 before logarithmic transformation. We used the R package mclust to implement Gaussian mixture modeling [36, 37] (specifically we used the following command: Mclust (logcount, G = 1:2, modelNames = “V”, prior = priorControl (shrinkage = 0.1, scale = 1))). Automatic model selection allowed us to detect amplicons for which all samples showed high (or low) counts since they were better modeled with a single Gaussian distribution. The use of log-transformed read counts allowed us to efficiently model the count data with Gaussian distributions (since the PCR amplification process is exponential) and this unsupervised classification scheme generally performed very efficiently. Specific instances of read count data, however, sometimes led to obvious classification errors and we therefore implemented two post hoc classification rules to automatically override genotypes obtained from Gaussian mixture fit of such data. First, amplicons for which all samples had very low or zero read counts were sometimes fitted with 2 Gaussian distributions instead of 1 or failed fitting for lack of data variability (resulting in numerical singularity), respectively. We thus set all genotype calls for a given amplicon to absent if all the samples had less than 10 specific reads. Second, single data clusters were sometimes wrongly fitted as 2 Gaussian distributions when a subset of the data incidentally had very low variance (so that a second distribution with artificially small standard deviation would be fitted to account for data heterogeneity). We thus automatically fitted the data with a single cluster when the automatic clustering resulted in cluster means that were less than 0.5 units (in log space) apart. Alternatively, we observed that setting a realistic prior on the variance (specifically, setting the scale parameter to 1) alleviated the need for the 2 post hoc rules and we used this strategy to perform genotype calls with the 104-loci libraries.

For amplicons fitted with 2 Gaussian distributions greater cluster separation was associated with higher calling confidence and we thus used the (log 10) odds ratio of the probability of belonging to the assigned cluster versus the other cluster as a genotyping quality score. We identified amplicons with failed clustering by plotting cluster means versus standard deviations and detecting outliers. For instance, excessive cluster standard deviation indicated failed separation (or inseparability) of the 2 clusters and such cases were dropped. Further, amplicons for which more than 80 % percent of the samples each had less than 80 % of their total number of tags uniquely aligned were also dropped because this pattern indicated that the sequence tags could be unspecific and therefore specificity of the amplicon to the targeted locus could not be insured. Following these two quality control steps, passed E amplicons were finally checked for miscalls due to the presence of very short L1 or Alu elements resulting in large numbers of specific reads. Thus, for each sample of each E amplicon, the genotype call was automatically set to absent (absent amplicon, i.e. L1 present) if the fraction of specific reads containing an L1Hs tag or AluYb8 tag was greater than 80 %.

Finally, allelic calls were made based on presence or absence calls for the E and G reactions, as described previously (Fig. 2b).

### Validation using individual site-specific PCR and gel electrophoresis

We performed individual site-specific PCR using the individual 5’- and 3’-flank primers for the E reactions and 3’- flank and L1Hs-or AluYb8-specific primers for the G reactions. Each 25 μl reaction consisted of 2× GoTaq Green Master Mix (Promega), 10 pmoles each of the two primers, and 100 ng of DNA. The thermal cycling protocol was 95 °C for 2 min, followed by 30 cycles of 95 °C for 30 s, 57 °C for 30 s, 73 °C for 1 min, and ended with 73 °C for 2 min. After the PCR, we visualized the products by running them on a 2 % TAE agarose gel.

### Phasing of heterozygous L1s using SNPs in library reads

We phased heterozygous L1s with the SNP data obtained from the 1000GP by calling known heterozygous SNPs in the sequencing reads. Specifically, for each particular L1, we first looked up SNPs previously discovered by the 1000GP (available at ftp://ftp.1000genomes.ebi.ac.uk/vol1/ftp/phase1/analysis_results/integrated_call_sets/ALL.chr1.integrated_phase1_v3.20101123.snps_indels_svs.genotypes.vcf.gz for chromosome 1 for instance) and that were located in the 3’ flank of the L1 (i.e. between the L1 3’ end and the 3’ flank primer). This sequence is covered by (the beginning of) read 1 in our sequencing libraries. Moreover, for heterozygous L1 samples, reads in the G and E libraries originate from the allele with and without the L1, respectively. We thus performed monoploid SNP genotype calls at the location of heterozygous SNPs identified in the 1000GP, in each of the E and G libraries. We used GATK [38] to call SNPs (specifically with “java -jar GenomeAnalysisTK.jar -R hg19.fa -T UnifiedGenotyper –L snptargetsfilename -ploidy 1 -dt NONE –I libfilename” where snptargetsfilename is a file with the location of the SNPs to be called and libfilename is a file containing aligned reads from either the E or the G library). We verified that the 2 SNP alleles called in the E and G libraries matched the 2 alleles recorded in the 1000GP data and assigned the L1 allele to either allele 1 or 2 depending on the match of the SNP allele called from the G library to the first or the second SNP allele recorded in the 1000GP data, respectively. If the 2 alleles called from the E and G libraries did not match the 2 alleles in the 1000GP data, we did not assign L1 phase. In the cases where there were several heterozygous SNPs in the L1 3’ flank, we phased the L1 based on each SNP independently and assigned the phase by a majority vote. We defined the phasing quality score as the fraction of heterozygous SNPs supporting the called phase over the total number of heterozygous SNPs. This quality score thus takes a (maximal) value of 1 if all heterozygous SNPs in the L1 flank support the same phase call.

### EHH analysis

EHH analyses were performed as previously described [14] using the final genotype and phase calls obtained for the 22-loci libraries obtained in two separate experiments (library preparation with the Access Array for 24 samples and manual library preparation for an additional 16 samples).

### Data access

The sequencing data are available from the Sequence Read Archive, accession SRP051735 (bioProject PRJNA271692).

## Additional files

Additional file 1: Table S1. Targeted L1 insertions, primers and library composition for the 60-loci L1 libraries (sheet “60 loci”), the 22-loci L1 libraries (sheet “22 loci”) and the 104-loci Alu libraries (sheet “104 loci”), as well the L1Hs-specific primer (G reaction for L1 libraries), the AluYb8-specific primer (G reaction for the Alu libraries) and the primers used for PCR 2 (sheet “Other primers”). L1 and Alu names correspond to the nomenclature used by the 1000GP [13]. Additional file 2: Figure S1. Gel electrophoresis analysis of the sequencing libraries following the second round PCR. a: Products of the 60-loci L1 libraries obtained for 12 HapMap samples. Primers were designed so that the E and G reactions span 200 bp (corresponding to final library products of 322 bp). b: Products of the 22-loci L1 libraries obtained for 24 HapMap samples. The primers were designed so that the E and G reactions span 400 bp (corresponding to final library products of 522 bp). c: Products of the 104-loci Alu libraries obtained for 40 HapMap samples. Primers were designed so that the E and G reactions span 200 bp (corresponding to final library products of 322 bp). Additional file 3: Figure S2. Computational pipeline for read count analysis and genotyping of L1 insertions. We depict the analysis of paired-end sequencing reads obtained from a hypothetical library targeting two L1 insertions. First, reads are sorted according to the primer sequences used to target each locus: the yellow and blue sequences represent the primers in the 3’, respectively 5’ flank of the first targeted L1 (locus 1) whereas the red and green sequences represent the primers in the 3’, respectively 5’ flank of the second targeted L1 (locus 2). The purple sequence represents the L1-specific primer. Upon sorting of every read into the 4 potential amplicons, we extracted 50-bp sequence tags immediately following the 3’ flank-primer sequence (gray box) and aligned them to the genome. We finally used the number of tags aligning to their targeted site as the basis for the presence/absence call for each of the E and G reactions. In addition, detection of L1 presence within reads originating from E reactions (not depicted here) is used to take into account the rare case of a very short L1 (see Methods). Additional file 4: Table S2. Read count-based genotype calls for the 60-loci libraries. The three Excel sheets (named “libE60”, “libG60”, and “lib60_allelic”) contain the genotype calls for the E libraries, G libraries and the allelic genotype calls, respectively. Additional file 5: Figure S3. Read counts, automatic genotype calls and validation results for the 22-loci libraries. a: Specific read counts for E reactions for 24 samples at each of 22 loci. Blue and black circles represent, respectively, the present and absent calls made based on the clustering of read counts. Crosses indicate genotypes with a quality score less than 7. Triangles indicate genotypes that would be called “present” (blue) because of high read count but that were called “absent” because the L1 sequence was detected in the reads (in the case of very short L1 insertions). b: Specific read counts obtained for E reactions for loci that passed quality control. Green and red circles indicate, respectively, concordant and discordant calls compared to the standard procedure using single-locus PCR reactions and gel electrophoresis. 5 genotype calls were discordant (loci 16 and 19). c: Same as a but for the G libraries. d: Same as b but for the G libraries. 1 genotype call was discordant (locus 19). Additional file 6: Figure S4. Read count cluster statistics and genotype quality scores for the 22-loci libraries. a: Mean versus standard deviation of clusters obtained with the E libraries. Black and blue circles indicate low and high read count clusters, respectively. Despite locus-to-locus variations, most clusters had similar means and standard deviations. Outliers represented loci that failed clustering. We manually set thresholds (gray lines) at 3 (mean) and 0.5 (standard deviation) and we dropped locus 5 (low read count cluster had mean greater than 3), and loci 10 and 15 (standard deviation greater than 0.5). b: same as a for G libraries, we dropped loci 8, 9, 12, 22 (standard deviation greater than 0.5). c: Histograms of genotype quality scores for the E libraries. Scores below 7 (threshold indicated by a gray vertical line) are indicated as crosses in Additional file 5: Figure S3a, c. d: Same as c for the G libraries. Additional file 7: Table S3. Read count-based genotype calls for the 22-loci libraries. The first four Excel sheets (named “libE22_24samples”, “libG22_24samples”, and “lib22_24samples_allelic” and “lib22_24samples_phase”) contain the genotype calls for the E libraries, G libraries, allelic genotype calls and phase calls (respectively) obtained for the initial 24 samples. 16 additional samples were genotyped in a separate experiment. Genotype and phase calls for these additional samples are provided in the last four Excel sheets (named “libE22_16samples”, “libG22_16samples”, and “lib22_16samples_allelic” and “lib22_16samples_phase”). Additional file 8: Figure S5. Gel electrophoresis of individual PCR reactions (validations) for the L1 insertions assayed with the 22-loci libraries. Additional file 9: Figure S6. Read counts, automatic genotype calls and comparison with calls obtained by the 1000 Genome Project (1000GP) for the 104-loci Alu libraries. a: Specific read counts for E reactions for 40 samples at each of 104 L1 loci. Blue and black circles represent, respectively, the present and absent calls made based on the clustering of read counts. Crosses indicate genotypes with a quality score less than 7. Triangles (locus 102) indicate genotypes that would be called “present” (blue) because of high read count but that were called “absent” because the Alu sequence was detected in the reads. b: Specific read counts obtained for E reactions for 88 loci that passed quality control. Green and purple circles indicate, respectively, concordant and discordant calls when comparing with genotype calls made by the 1000GP. Black circles represent calls that were assigned a low quality by the 1000GP and that we did not use in the comparison. 68 genotypes calls were discordant. The complete list and validation of discordant calls using individual PCR reactions are shown in Additional file 12: Figure S8 . c: Same as a but for the G libraries. d: Same as b for 96 loci that passed quality control. 84 genotypes calls were discordant. The complete list and validation of discordant calls using individual PCR reactions are shown in Additional file 12: Figure S8. Additional file 10: Figure S7. Read count cluster statistics and genotype quality scores for the 104-loci Alu libraries. a: Mean versus standard deviation of clusters obtained with the E libraries. Black and blue circles indicate, respectively, low and high read count clusters. Despite locus-to-locus variations, most clusters had similar means and standard deviations. We manually set thresholds (represented as gray lines) at 2 (mean) and 0.6 (standard deviation), which dropped out loci 13, 17, 19, 39, 62, 80, 82, 93, 96 and 99 (high read count cluster had mean less than 2) and locus 53 (standard deviation greater than 0.6). b: Same as a for the G libraries. We manually set 2 thresholds on the cluster mean (represented as gray lines) at 1.5 (dropping loci with low count cluster greater than 1.5) and 2 (dropping loci with high count cluster less than 2). As a result, we dropped locus 53 (low read count cluster with mean greater than 1.5) and loci 39, 62, 63, 80 and 99 (high read count cluster with mean less than 2). c: Histograms of genotype quality scores obtained for the E libraries. Scores below 7 (threshold indicated by a gray vertical line) are indicated as crosses in Additional file 9: Figure S6a, c. d: Same as c for the G libraries. Additional file 11: Table S4. Read count-based genotype calls for the 104-loci Alu libraries. The three Excel sheets (named “libE104”, “libG104”, and “lib104_allelic”) contain the genotype calls for the E libraries, G libraries and the allelic genotype calls, respectively. Additional file 12: Figure S8. Gel electrophoresis of individual PCR reactions (validations) for the Alu genotype calls that were found to be discordant with the calls made by the 1000GP. Additional file 13: Table S5. Material and reagent costs for genotyping experiments of various scales (a: 48 libraries and 48 samples, b: 24 libraries and 24 samples, c: 12 libraries and 12 samples). Three methods are compared: automated library preparation followed by sequencing, manual library preparation followed by sequencing and standard, individual PCR followed by gel electrophoresis. Costs for primer synthesis and labour costs are not taken into consideration. Prices are in Singapore dollars before taxes. 1 Singapore dollar corresponds approximately to 0.8 US dollars.

## Abbreviations

TE: Transposable element
L1: LINE-1 retrotransposon
SNP: Single nucleotide polymorphism
