# Supplementary figures and images for: Read count-based method for high-throughput allelic genotyping of transposable elements and structural variants
Source: BMC Genomics. 2015 Jul 8;16(1):508. doi: 10.1186/s12864-015-1700-4 (PMC4494700; doi:10.1186/s12864-015-1700-4)

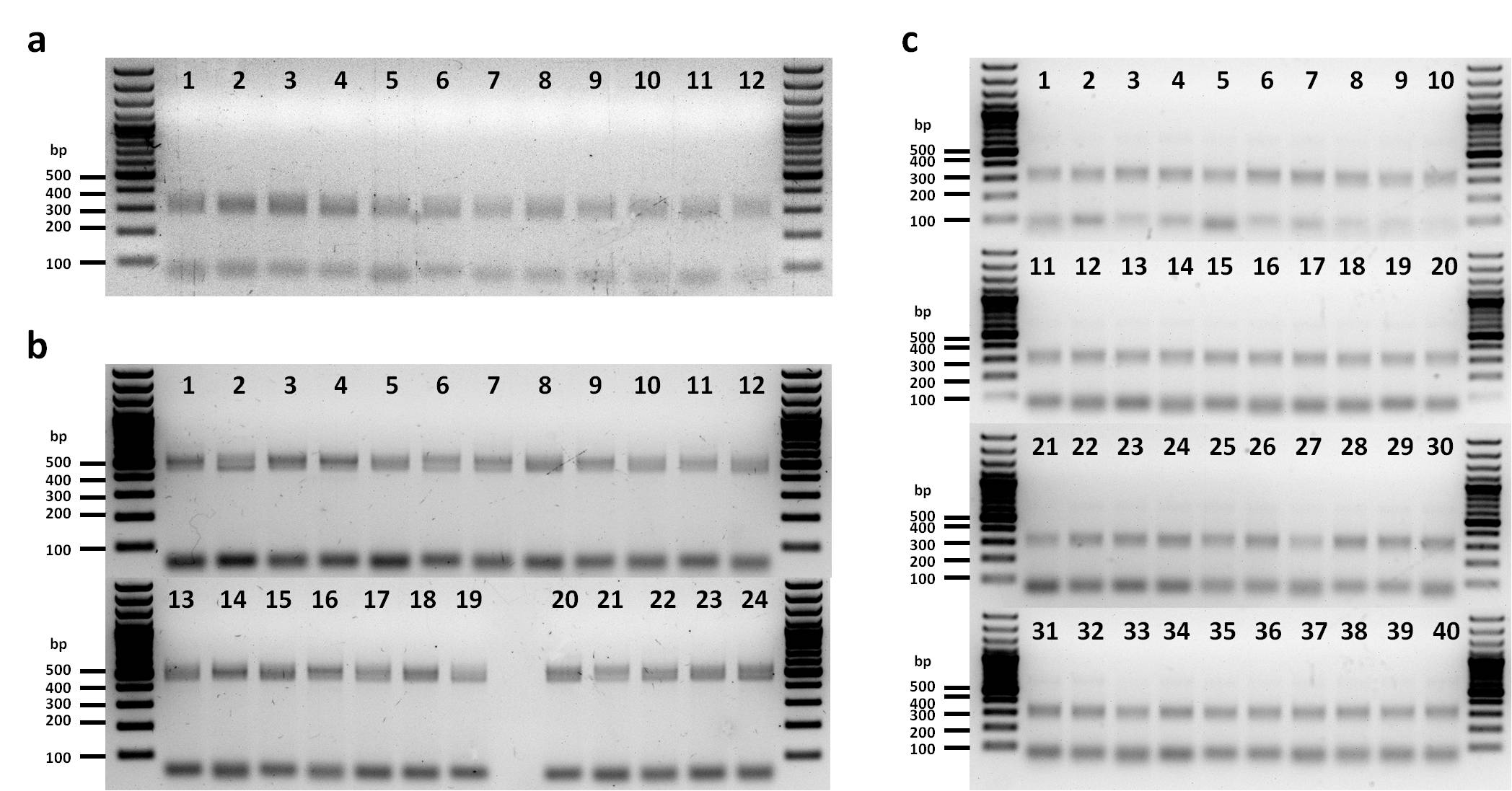

Supplement: Additional file 2: Figure S1. — Gel electrophoresis analysis of the sequencing libraries following the second round PCR. a: Products of the 60-loci L1 libraries obtained for 12 HapMap samples. Primers were designed so that the E and G reactions span 200 bp (corresponding to final library products of 322 bp). b: Products of the 22-loci L1 libraries obtained for 24 HapMap samples. The primers were designed so that the E and G reactions span 400 bp (corresponding to final library products of 522 bp). c: Products of the 104-loci Alu libraries obtained for 40 HapMap samples. Primers were designed so that the E and G reactions span 200 bp (corresponding to final library products of 322 bp). [file 12864_2015_1700_MOESM2_ESM.jpg]

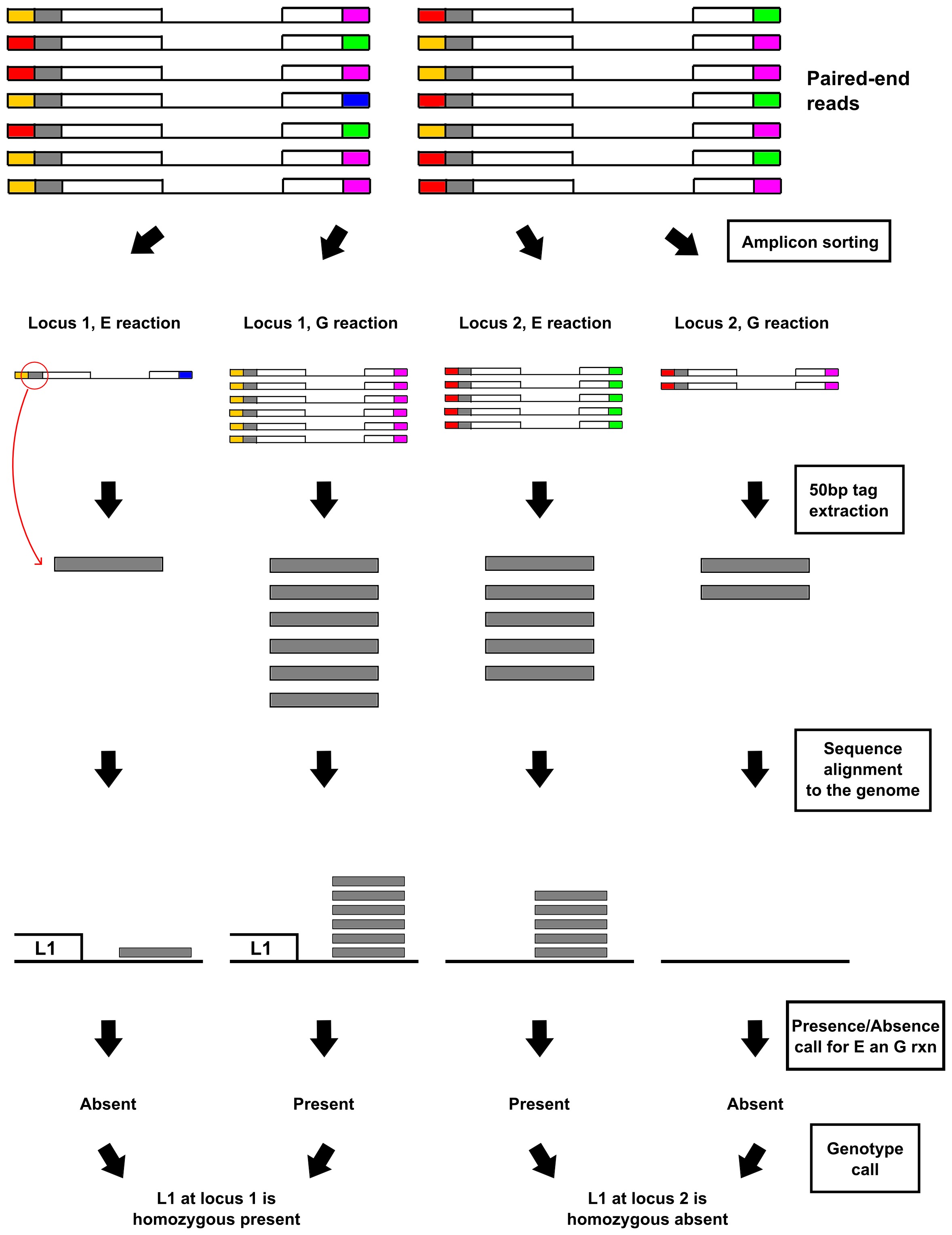

Supplement: Additional file 3: Figure S2. — Computational pipeline for read count analysis and genotyping of L1 insertions. We depict the analysis of paired-end sequencing reads obtained from a hypothetical library targeting two L1 insertions. First, reads are sorted according to the primer sequences used to target each locus: the yellow and blue sequences represent the primers in the 3’, respectively 5’ flank of the first targeted L1 (locus 1) whereas the red and green sequences represent the primers in the 3’, respectively 5’ flank of the second targeted L1 (locus 2). The purple sequence represents the L1-specific primer. Upon sorting of every read into the 4 potential amplicons, we extracted 50-bp sequence tags immediately following the 3’ flank-primer sequence (gray box) and aligned them to the genome. We finally used the number of tags aligning to their targeted site as the basis for the presence/absence call for each of the E and G reactions. In addition, detection of L1 presence within reads originating from E reactions (not depicted here) is used to take into account the rare case of a very short L1 (see Methods). [file 12864_2015_1700_MOESM3_ESM.jpg]

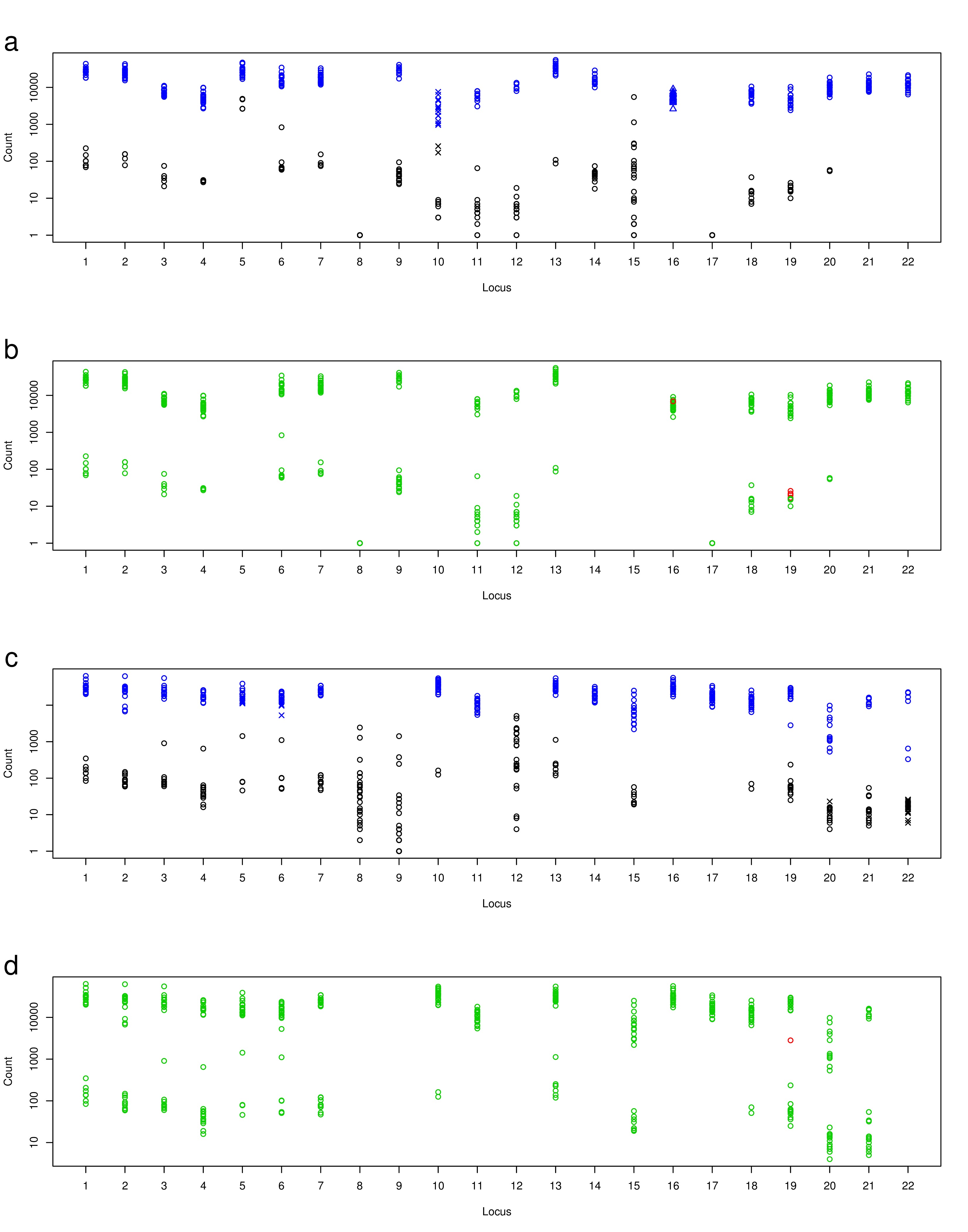

Supplement: Additional file 5: Figure S3. — Read counts, automatic genotype calls and validation results for the 22-loci libraries. a: Specific read counts for E reactions for 24 samples at each of 22 loci. Blue and black circles represent, respectively, the present and absent calls made based on the clustering of read counts. Crosses indicate genotypes with a quality score less than 7. Triangles indicate genotypes that would be called “present” (blue) because of high read count but that were called “absent” because the L1 sequence was detected in the reads (in the case of very short L1 insertions). b: Specific read counts obtained for E reactions for loci that passed quality control. Green and red circles indicate, respectively, concordant and discordant calls compared to the standard procedure using single-locus PCR reactions and gel electrophoresis. 5 genotype calls were discordant (loci 16 and 19). c: Same as a but for the G libraries. d: Same as b but for the G libraries. 1 genotype call was discordant (locus 19). [file 12864_2015_1700_MOESM5_ESM.jpg]

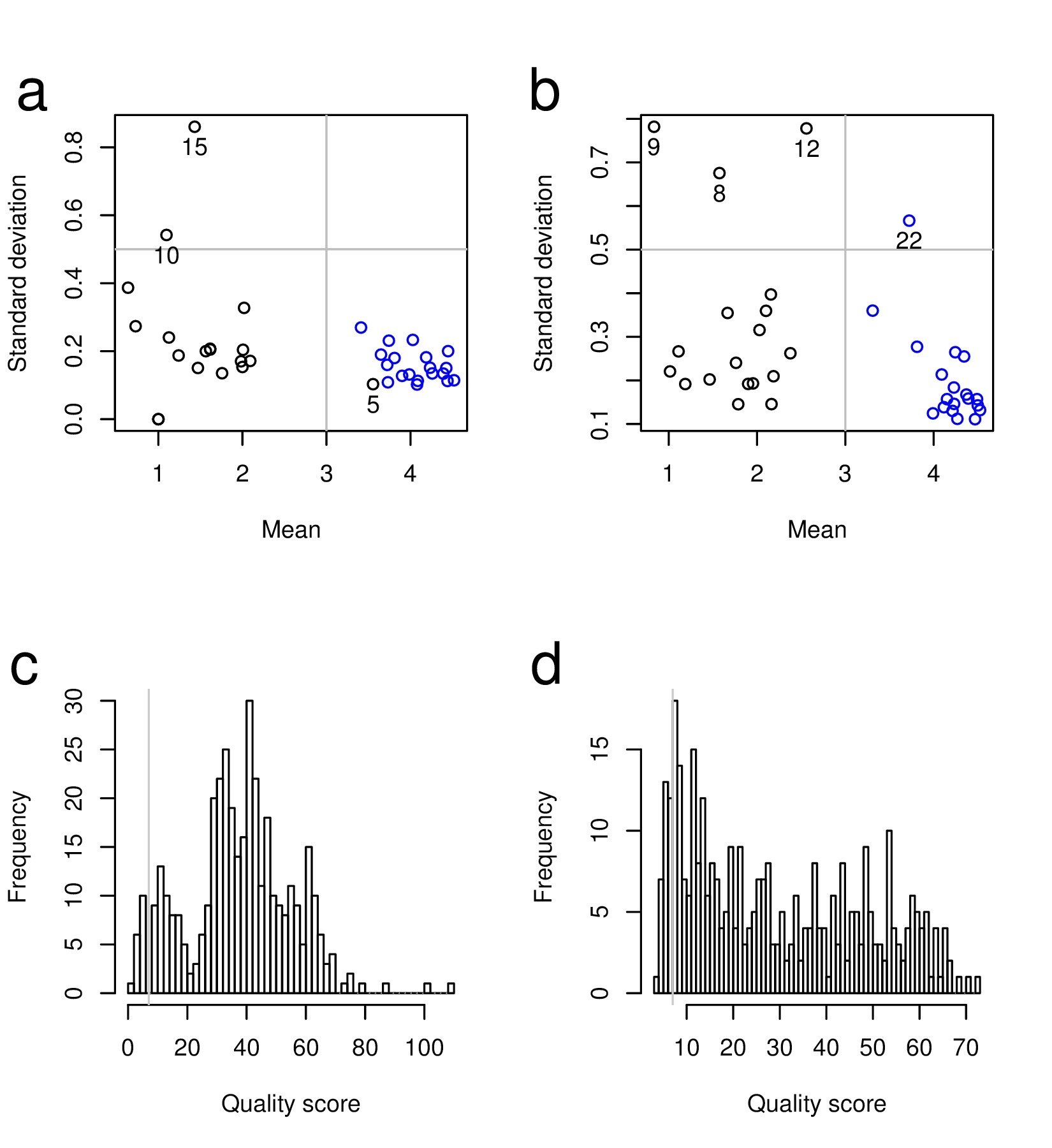

Supplement: Additional file 6: Figure S4. — Read count cluster statistics and genotype quality scores for the 22-loci libraries. a: Mean versus standard deviation of clusters obtained with the E libraries. Black and blue circles indicate low and high read count clusters, respectively. Despite locus-to-locus variations, most clusters had similar means and standard deviations. Outliers represented loci that failed clustering. We manually set thresholds (gray lines) at 3 (mean) and 0.5 (standard deviation) and we dropped locus 5 (low read count cluster had mean greater than 3), and loci 10 and 15 (standard deviation greater than 0.5). b: same as a for G libraries, we dropped loci 8, 9, 12, 22 (standard deviation greater than 0.5). c: Histograms of genotype quality scores for the E libraries. Scores below 7 (threshold indicated by a gray vertical line) are indicated as crosses in Additional file 5: Figure S3a, c. d: Same as c for the G libraries. [file 12864_2015_1700_MOESM6_ESM.jpg]

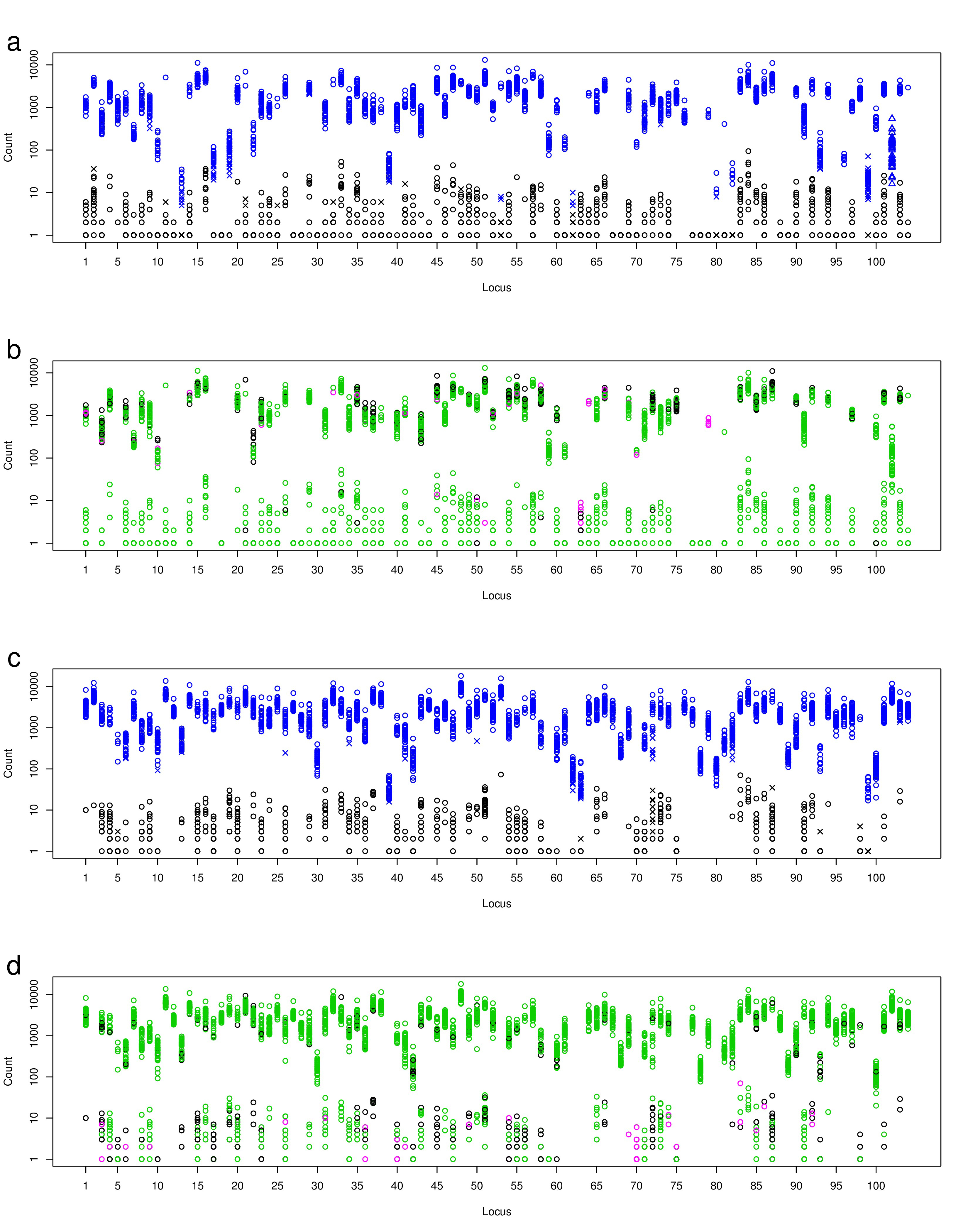

Supplement: Additional file 9: Figure S6. — Read counts, automatic genotype calls and comparison with calls obtained by the 1000 Genome Project (1000GP) for the 104-loci Alu libraries. a: Specific read counts for E reactions for 40 samples at each of 104 L1 loci. Blue and black circles represent, respectively, the present and absent calls made based on the clustering of read counts. Crosses indicate genotypes with a quality score less than 7. Triangles (locus 102) indicate genotypes that would be called “present” (blue) because of high read count but that were called “absent” because the Alu sequence was detected in the reads. b: Specific read counts obtained for E reactions for 88 loci that passed quality control. Green and purple circles indicate, respectively, concordant and discordant calls when comparing with genotype calls made by the 1000GP. Black circles represent calls that were assigned a low quality by the 1000GP and that we did not use in the comparison. 68 genotypes calls were discordant. The complete list and validation of discordant calls using individual PCR reactions are shown in Additional file 12: Figure S8 . c: Same as a but for the G libraries. d: Same as b for 96 loci that passed quality control. 84 genotypes calls were discordant. The complete list and validation of discordant calls using individual PCR reactions are shown in Additional file 12: Figure S8. [file 12864_2015_1700_MOESM9_ESM.jpg]

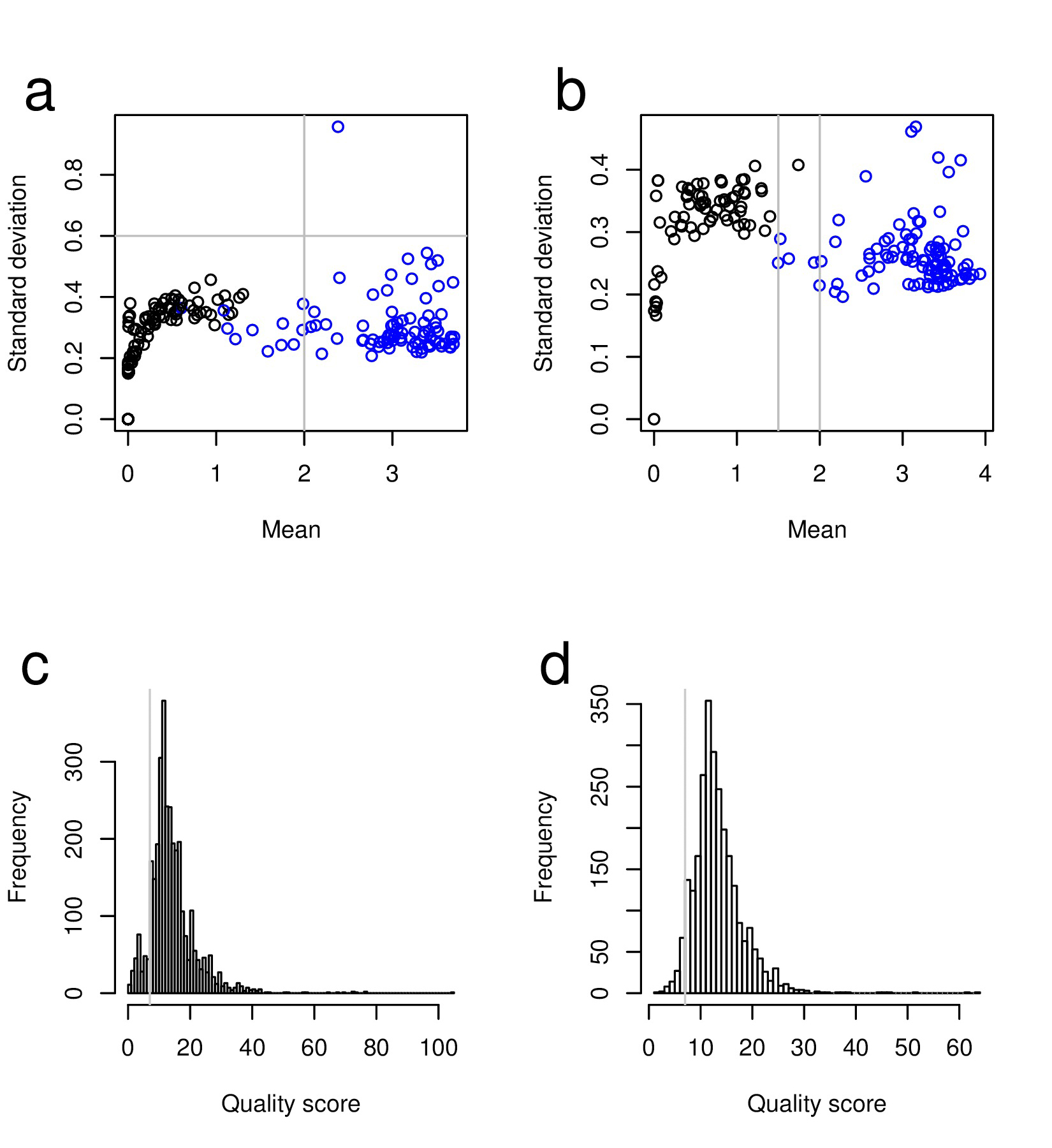

Supplement: Additional file 10: Figure S7. — Read count cluster statistics and genotype quality scores for the 104-loci Alu libraries. a: Mean versus standard deviation of clusters obtained with the E libraries. Black and blue circles indicate, respectively, low and high read count clusters. Despite locus-to-locus variations, most clusters had similar means and standard deviations. We manually set thresholds (represented as gray lines) at 2 (mean) and 0.6 (standard deviation), which dropped out loci 13, 17, 19, 39, 62, 80, 82, 93, 96 and 99 (high read count cluster had mean less than 2) and locus 53 (standard deviation greater than 0.6). b: Same as a for the G libraries. We manually set 2 thresholds on the cluster mean (represented as gray lines) at 1.5 (dropping loci with low count cluster greater than 1.5) and 2 (dropping loci with high count cluster less than 2). As a result, we dropped locus 53 (low read count cluster with mean greater than 1.5) and loci 39, 62, 63, 80 and 99 (high read count cluster with mean less than 2). c: Histograms of genotype quality scores obtained for the E libraries. Scores below 7 (threshold indicated by a gray vertical line) are indicated as crosses in Additional file 9: Figure S6a, c. d: Same as c for the G libraries. [file 12864_2015_1700_MOESM10_ESM.jpg]
