# Supplementary material for: Read count-based method for high-throughput allelic genotyping of transposable elements and structural variants
Source: BMC Genomics. 2015 Jul 8;16(1):508. doi: 10.1186/s12864-015-1700-4 (PMC4494700; doi:10.1186/s12864-015-1700-4)
Supplement: Additional file 8: Figure S5. — Gel electrophoresis of individual PCR reactions (validations) for the L1 insertions assayed with the 22-loci libraries. [file 12864_2015_1700_MOESM8_ESM.pdf]

Gel electrophoresis of individual PCR reactions for L1 insertions assayed with the 22-loci libraries

L1 name	Comment	Page
P1_M_061510_1_185		2
P1_M_061510_1_239		3
P1_M_061510_1_391		4
P1_M_061510_4_203		5
P1_M_061510_9_218		6
P1_M_061510_10_203		7
P1_M_061510_3_279		8
P1_M_061510_1_131		9
P1_M_061510_1_131	Additional 24 HapMap samples[a]	10
P1_M_061510_3_279		11
P1_M_061510_3_293		12
P1_M_061510_6_823		13
P1_M_061510_7_376		14

L1 name	Comment	Page
P1_M_061510_8_220		15
P1_M_061510_8_220	Redesigned G primer[b]	16
P1_M_061510_10_299		17
P1_M_061510_13_47		18
P1_M_061510_14_175		19
P1_M_061510_14_175	Redesigned G primer[c]	20
P1_M_061510_18_386		21
P1_M_061510_18_386	Additional 24 HapMap samples[d]	22
P1_M_061510_20_28		23
P1_M_061510_20_89		24
P1_M_061510_20_89	Redesigned G primer[e]	25
P1_MEI_190&P2_MEI_1442		26
P1_MEI_2893&P2_MEI_1141		27
P1_MEI_3120		28

[a] We tested additional HapMap samples to find heterozygous samples and verify that the E reaction worked (see e.g. NA12749).
[b] Additional validation for P1_M_061510_8_220 using a redesigned (G) primer. The original primers generated unspecific products (for reaction G) and did not allow for unambiguous genotyping by gel electrophoresis.
[c] Additional validation for P1_M_061510_14_175 using a redesigned (G) primer that does not overlap any known SNPs. For the E reaction with NA12003, the lower band (allele without L1) now shows higher abundance compared to the higher band (allele with L1).
[d] Same as a but for P1_M_061510_18_386 (see e.g. NA12155)
[e] Additional validation for P1_M_061510_20_89 using a redesigned (G) primer. The original primers generated unspecific products (in particular for reaction E) and did not allow for genotyping by gel electrophoresis.

P1_M_061510_1_185

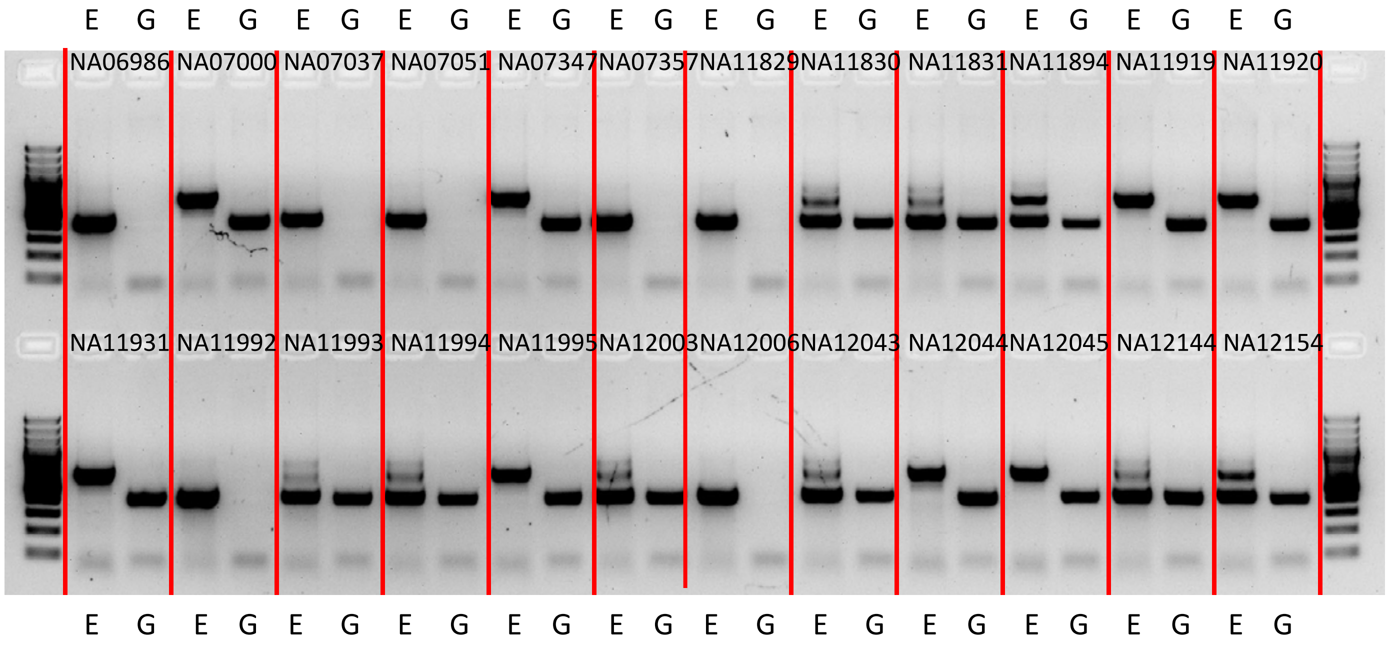

P1_M_061510_1_239

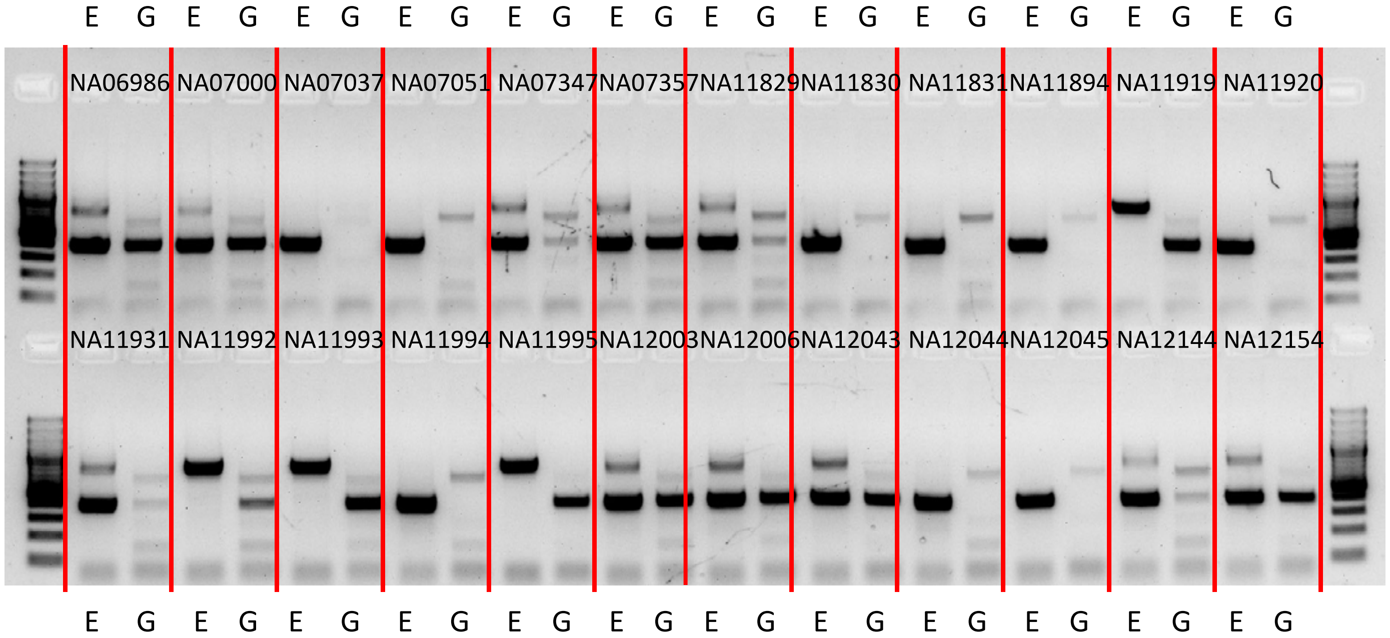

P1_M_061510_1_391

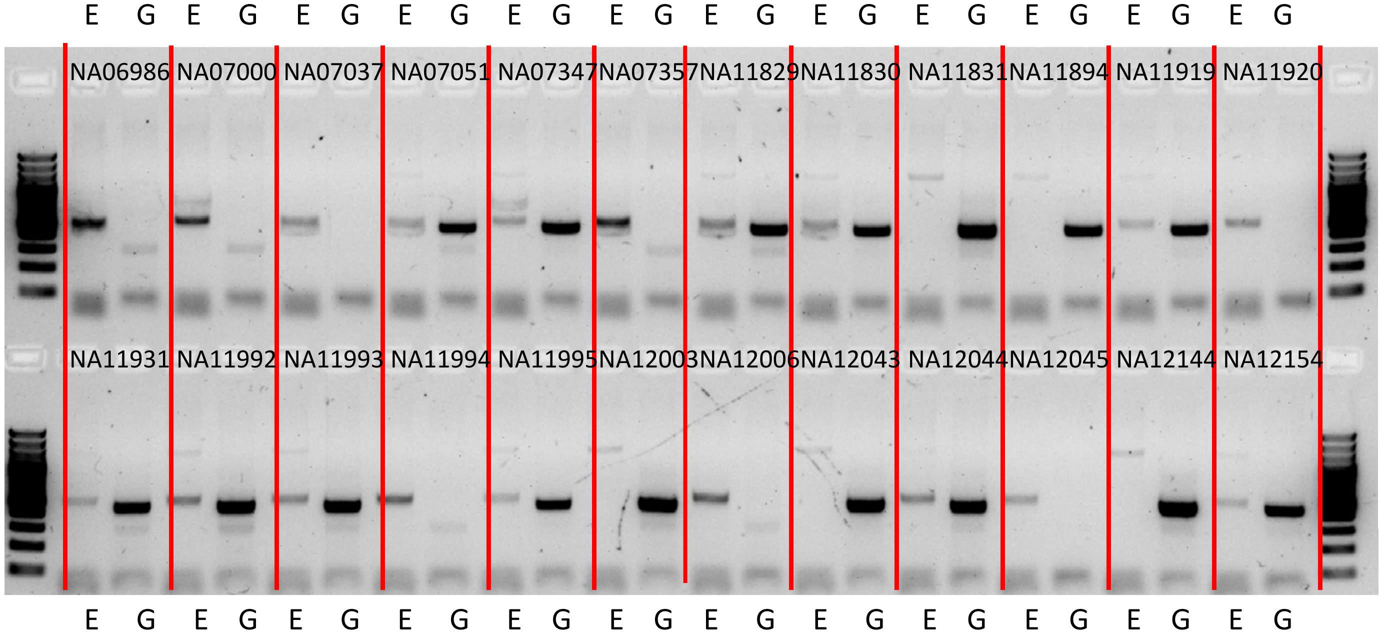

P1_M_061510_4_203

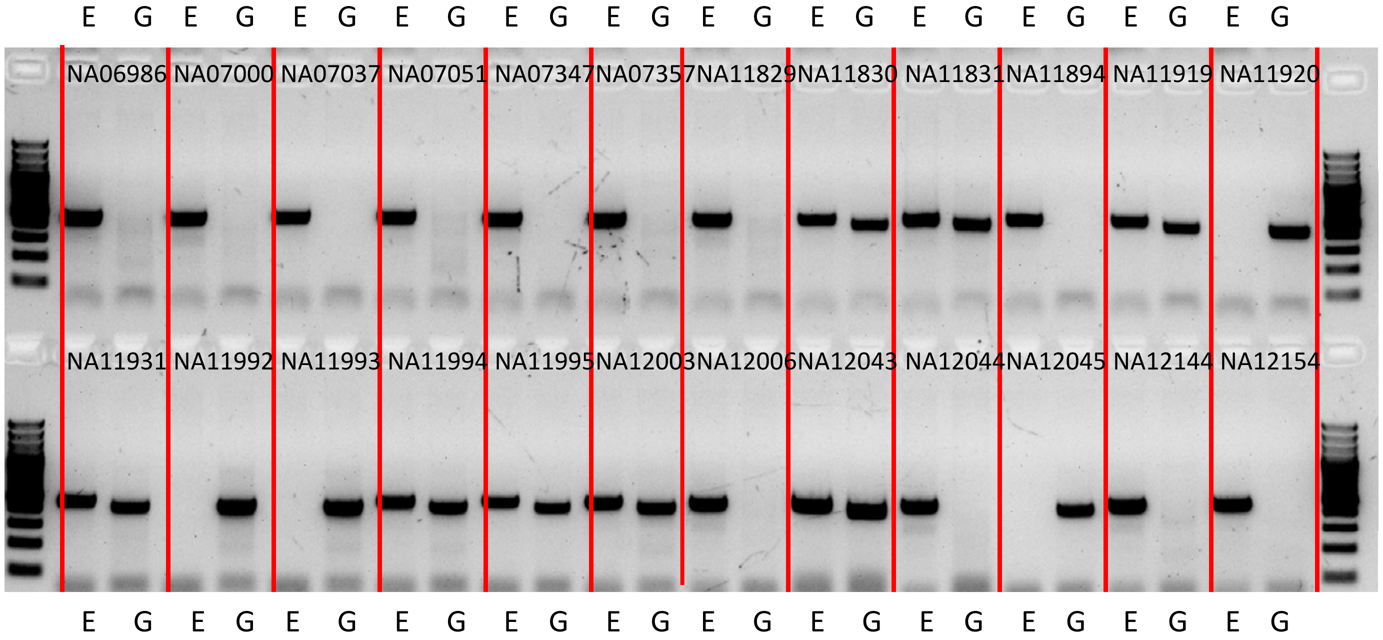

P1_M_061510_9_218

E G E G E G E G E G E G E G E G E G E G E G E G

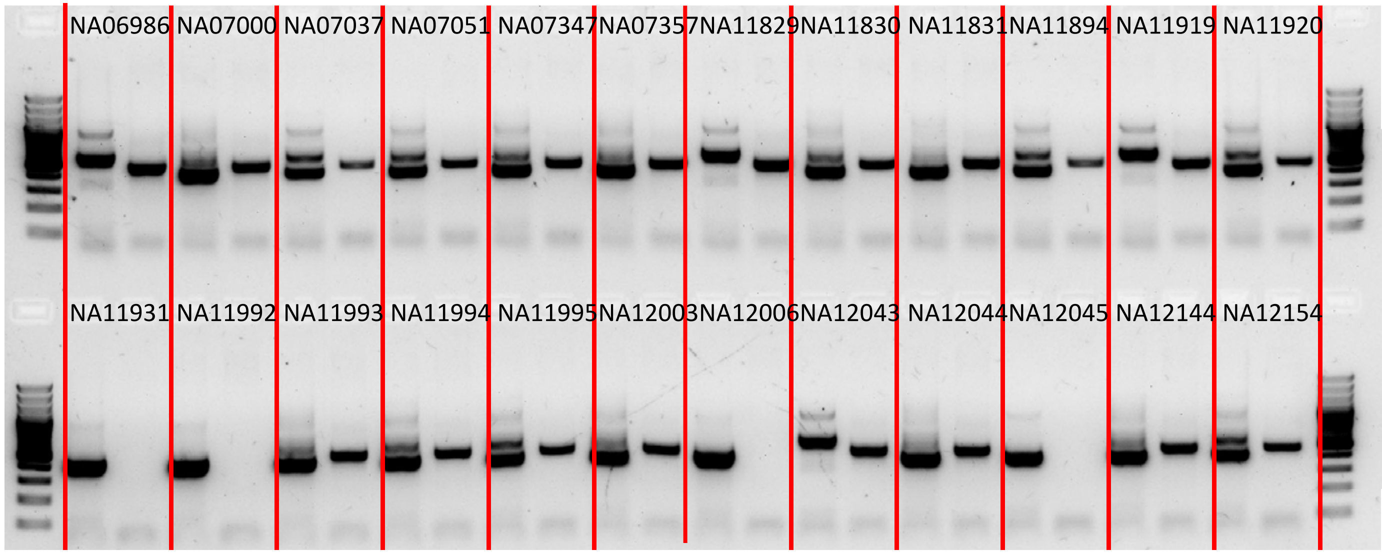

E G E G E G E G E G E G E G E G E G E G E G E G

P1_M_061510_10_203

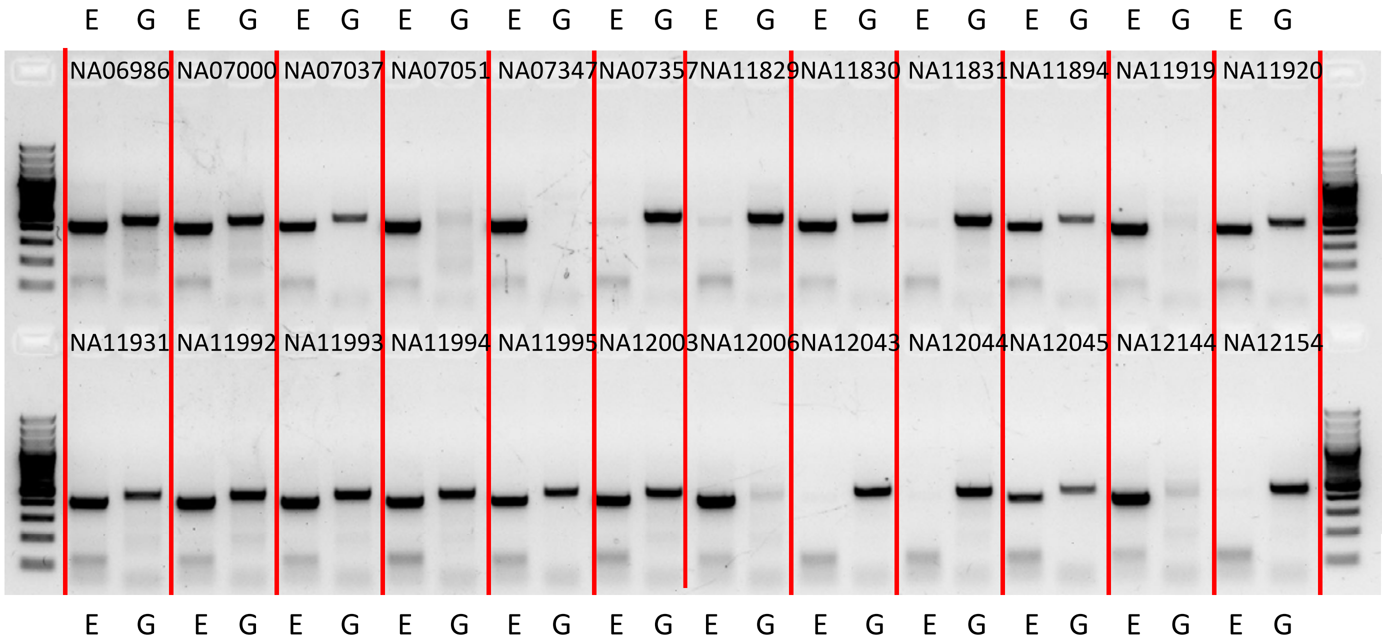

P1_MEI_1280&P2_MEI_1388

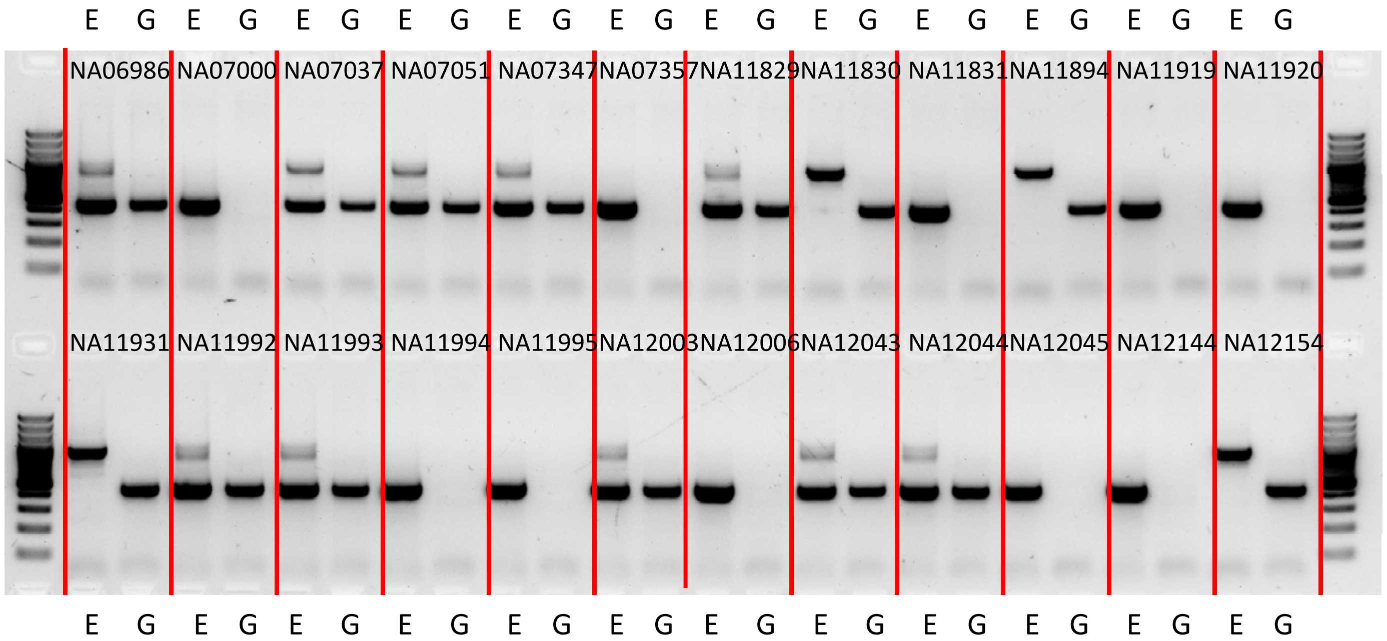

P1_M_061510_1_131

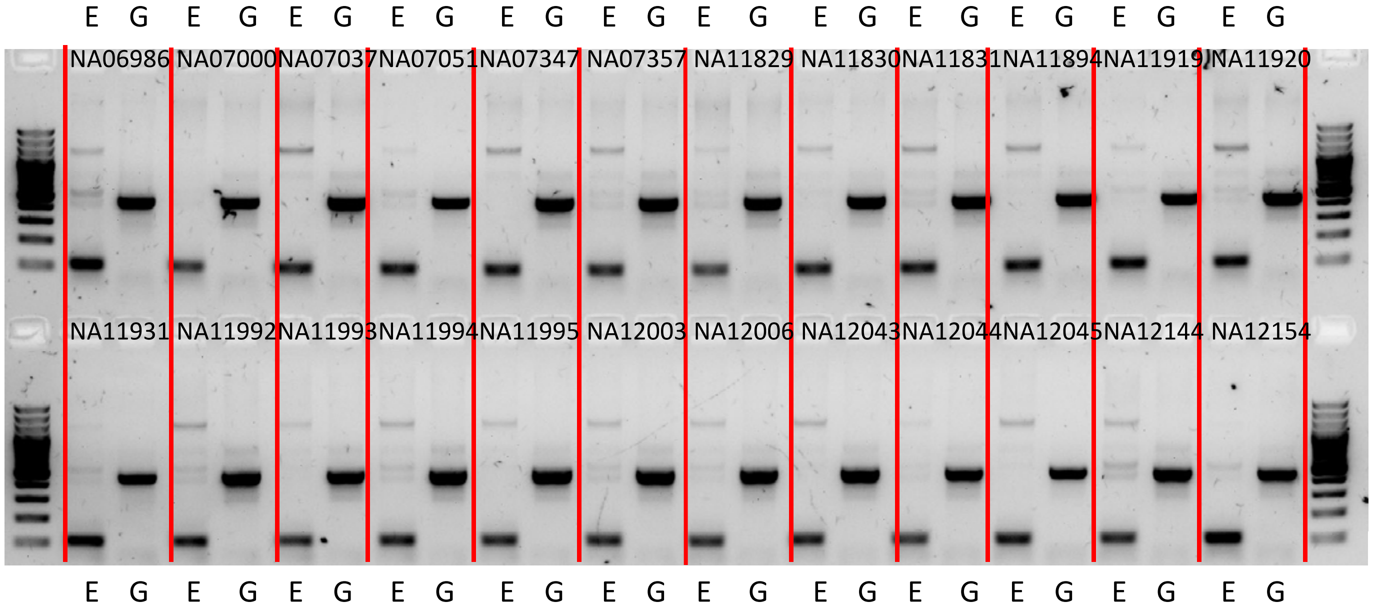

P1_M_061510_1_131

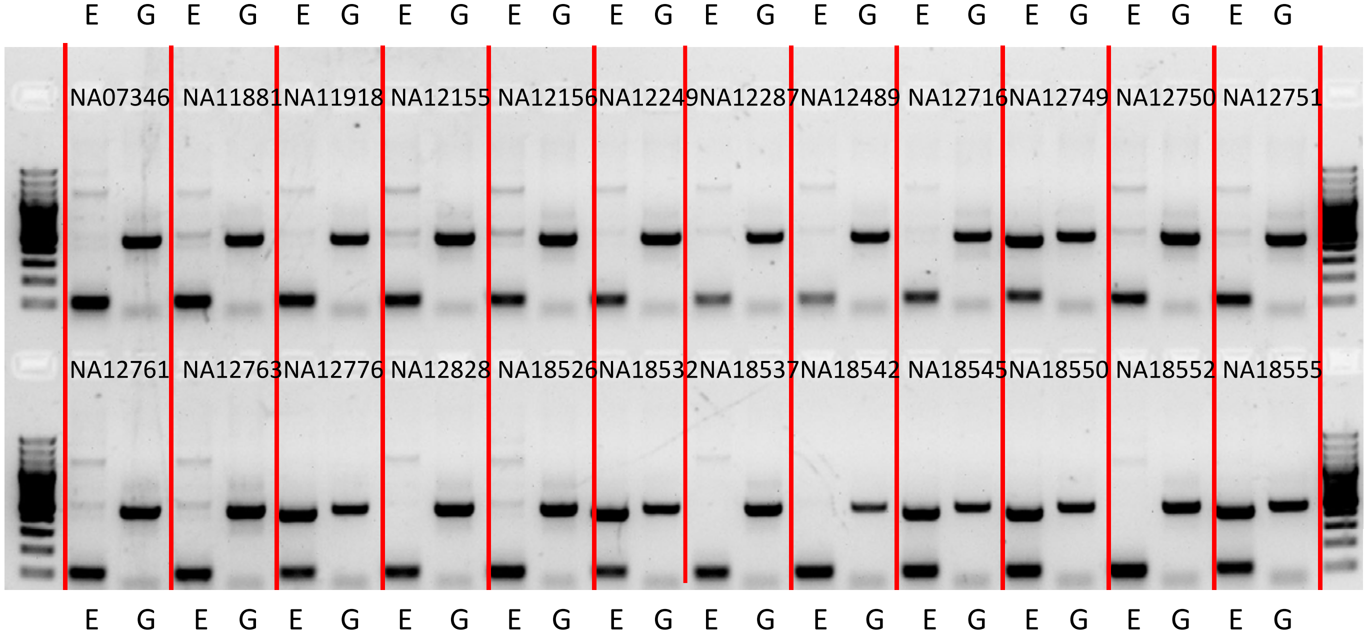

P1_M_061510_3_279

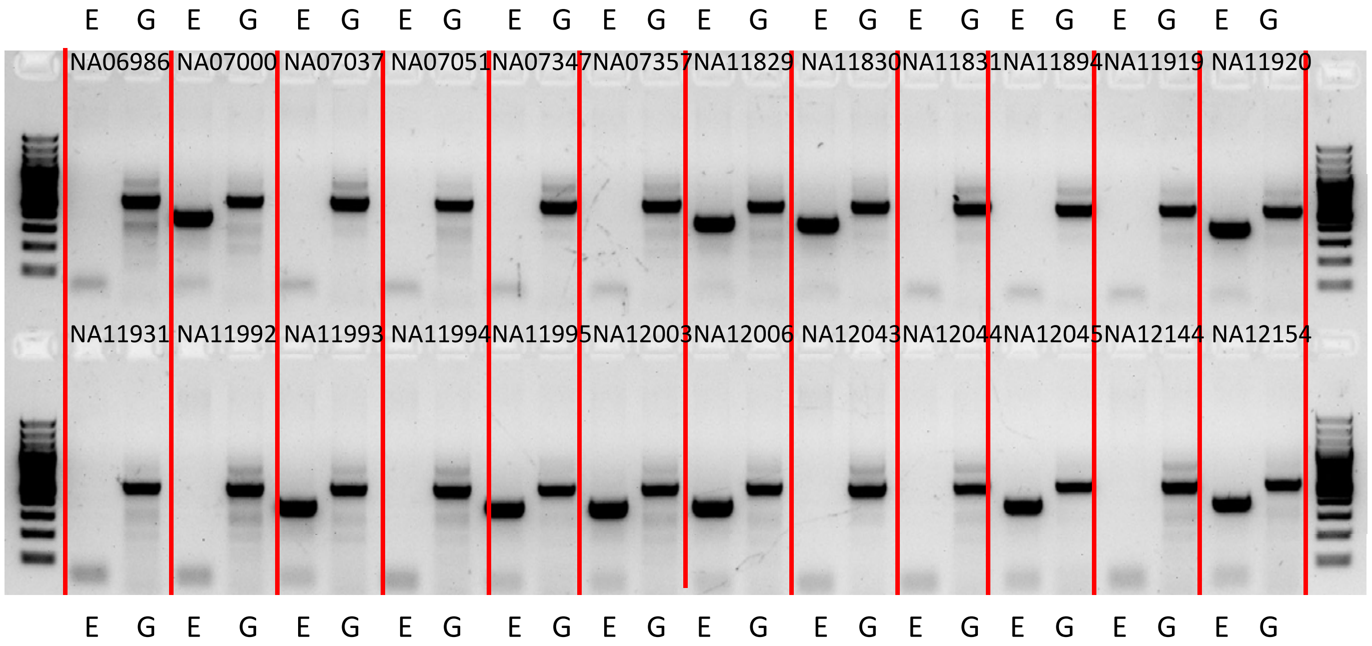

P1_M_061510_3_293

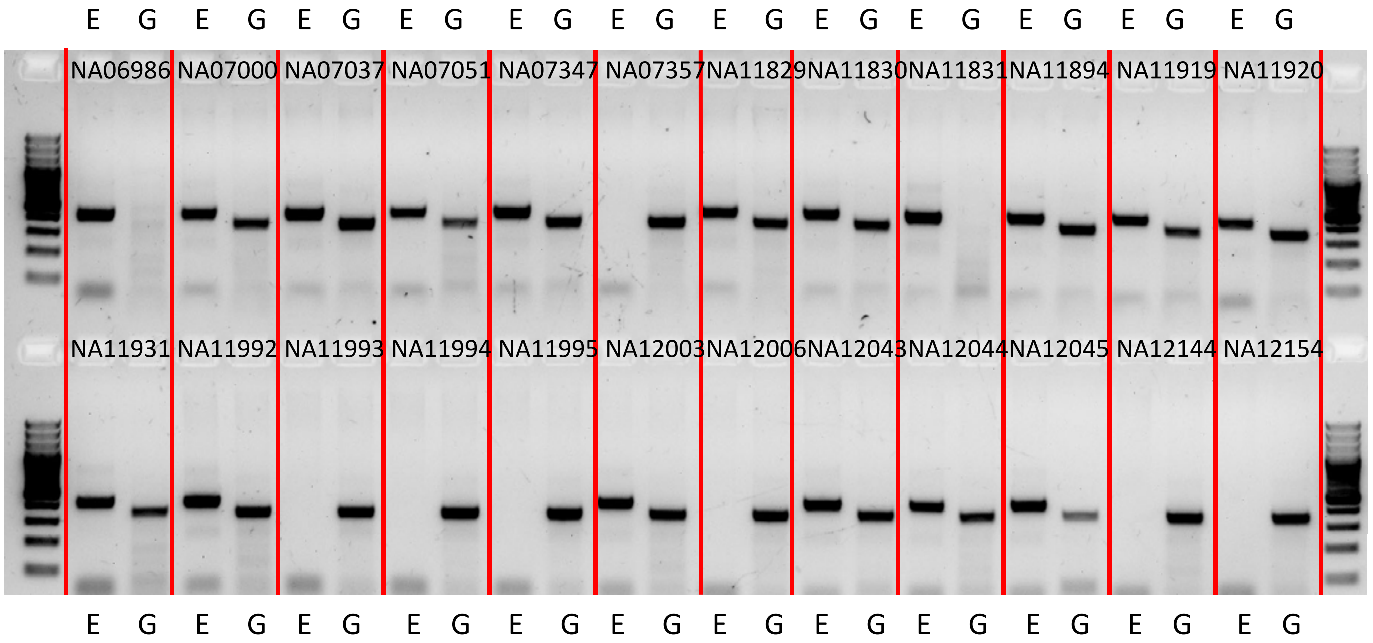

P1_M_061510_6_823

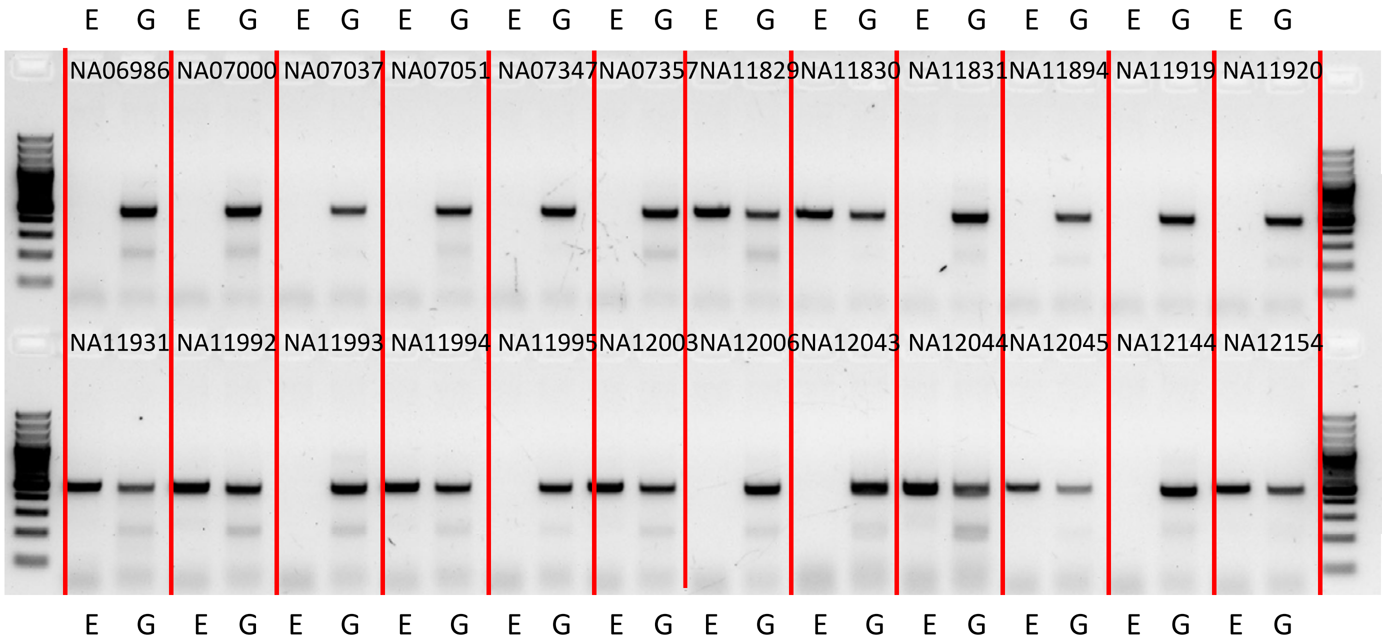

P1_M_061510_7_376

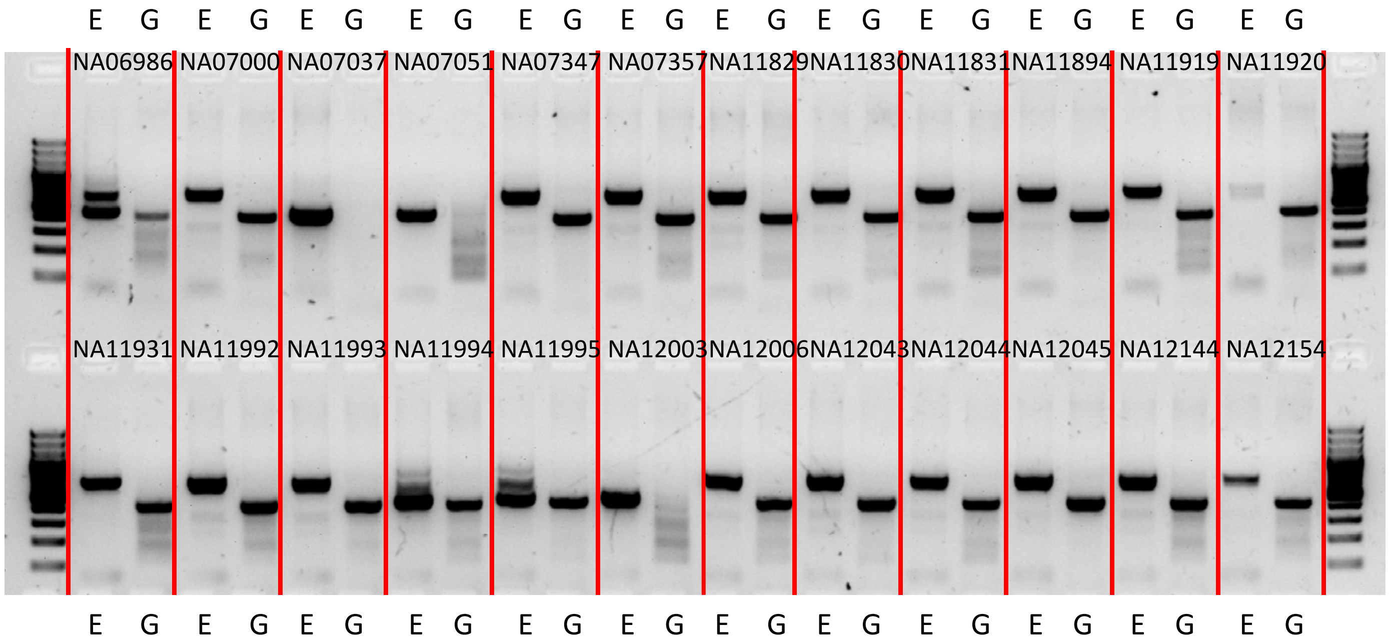

P1_M_061510_8_220

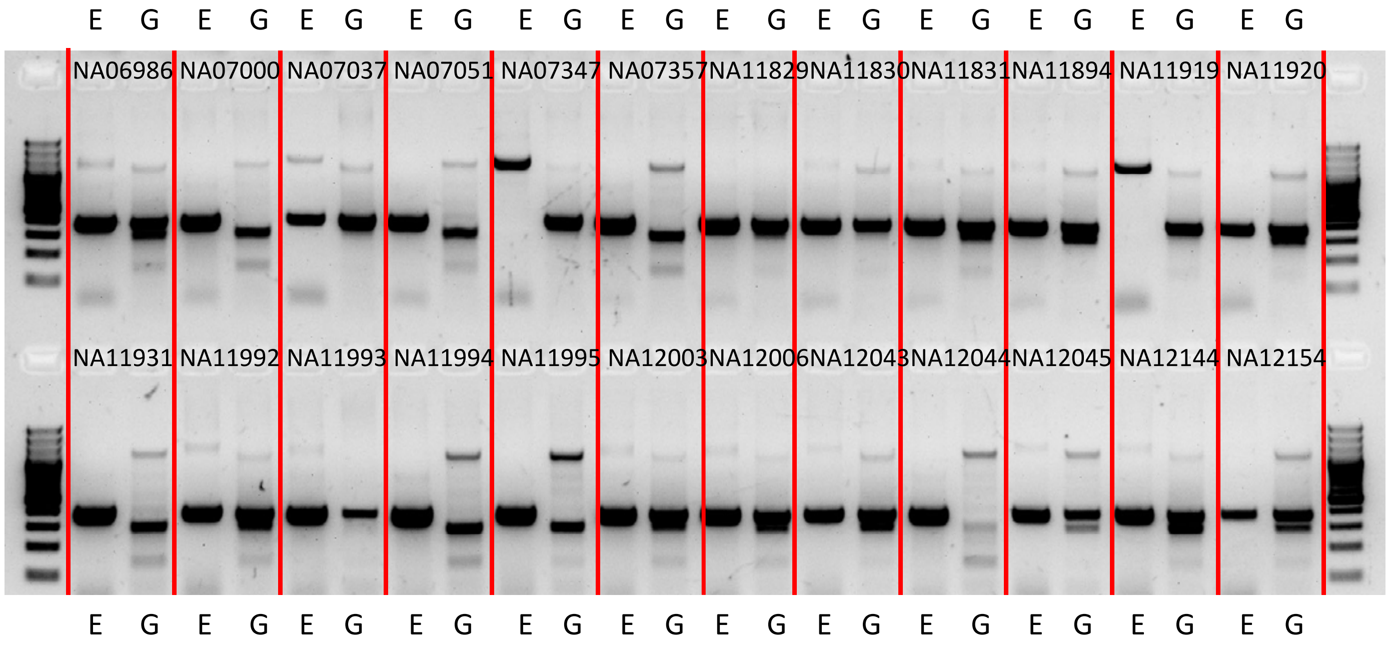

P1_M_061510_8_220

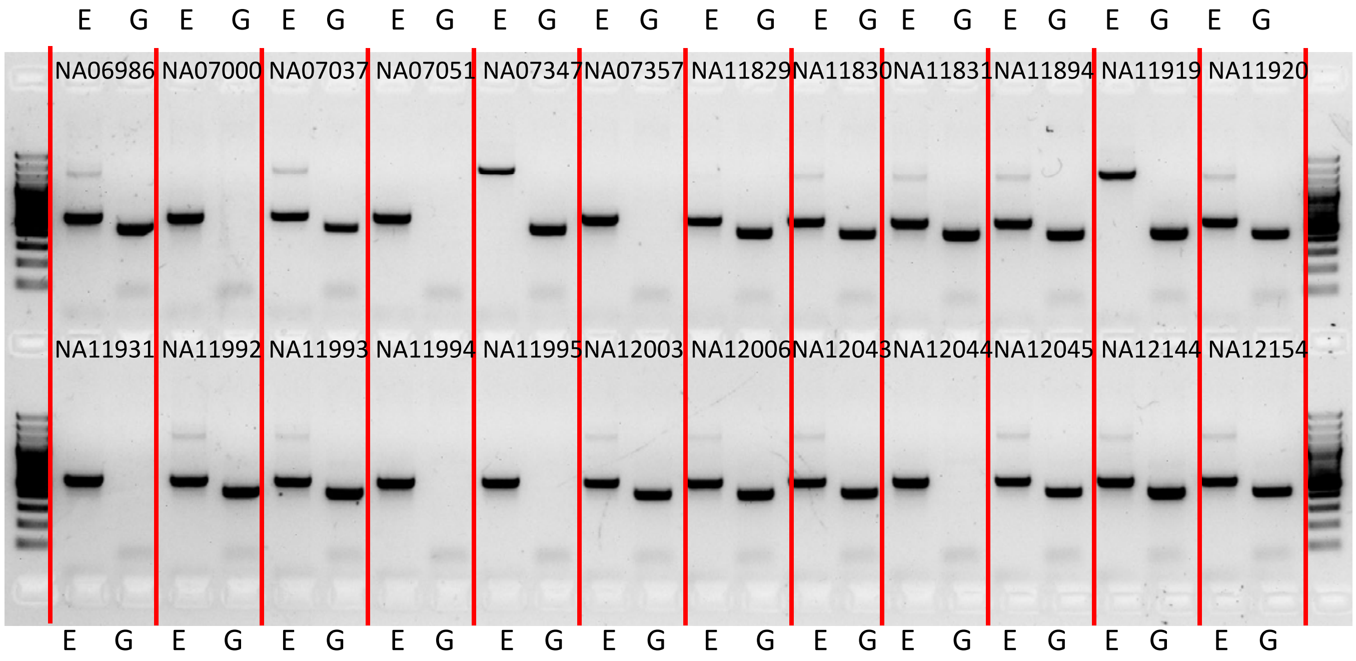

P1_M_061510_10_299

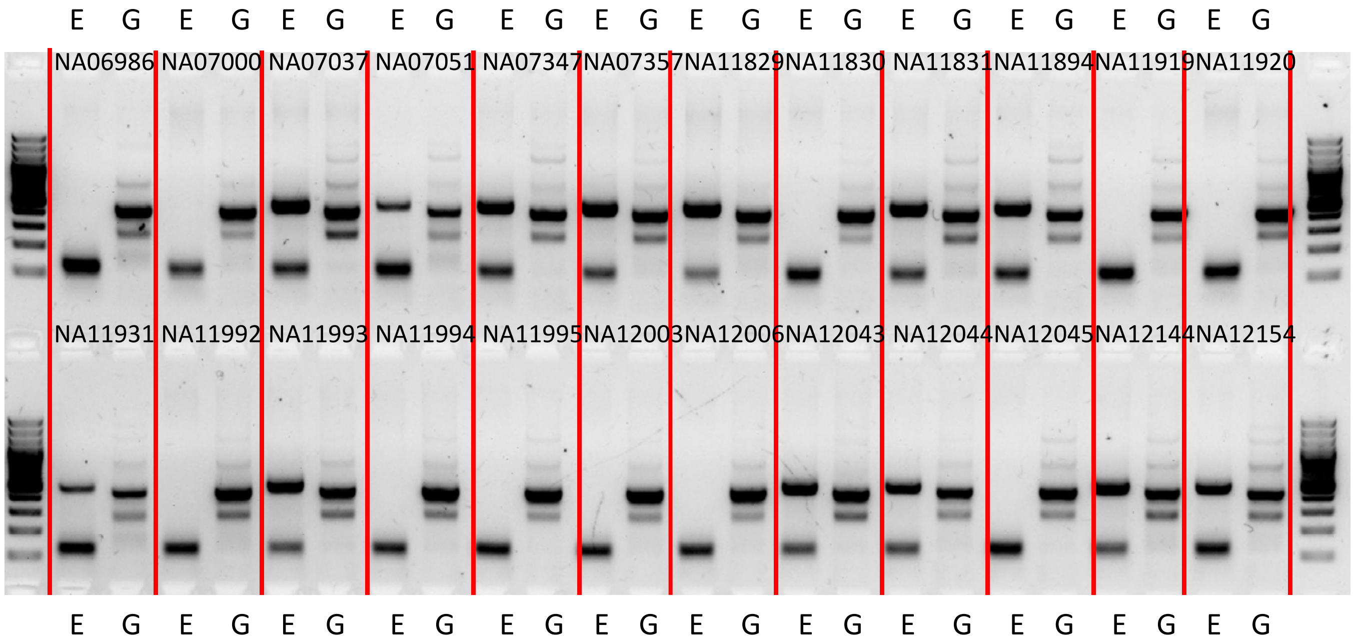

P1_M_061510_13_47

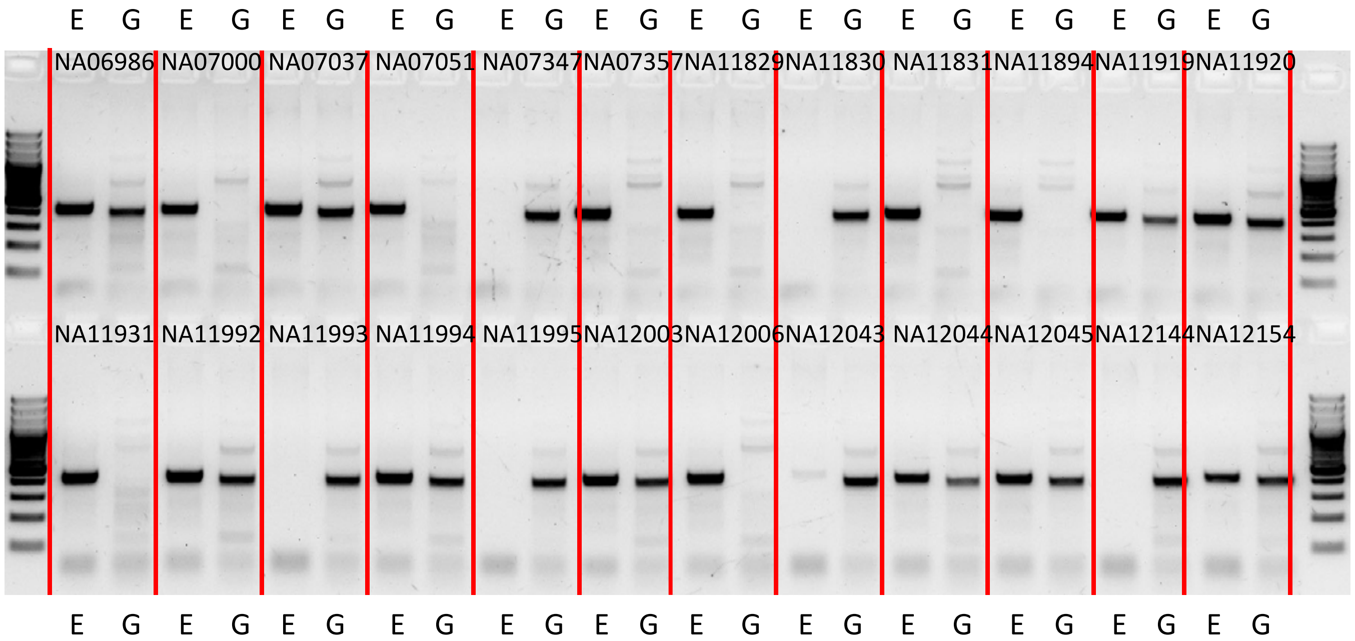

P1_M_061510_14_175

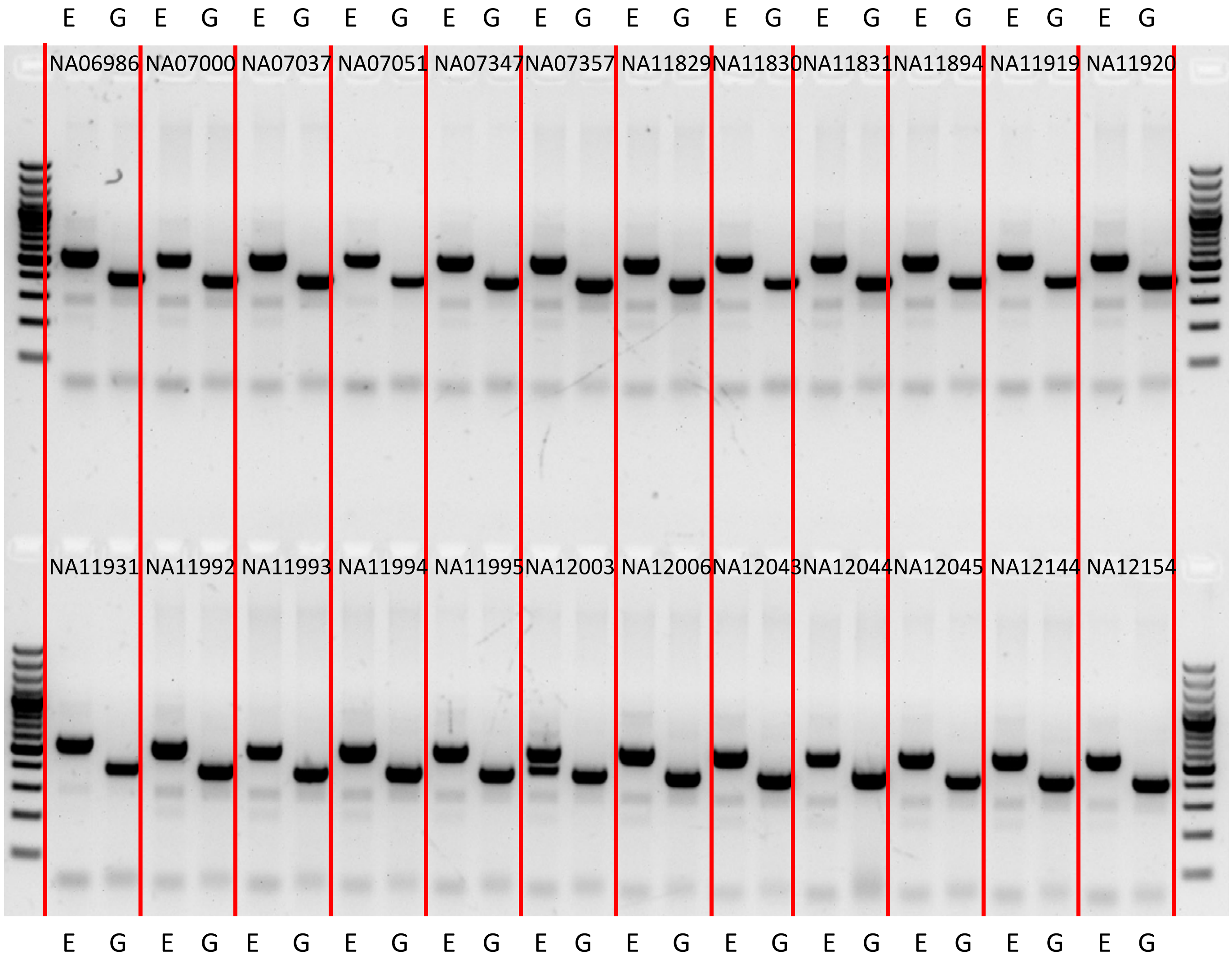

P1_M_061510_14_175

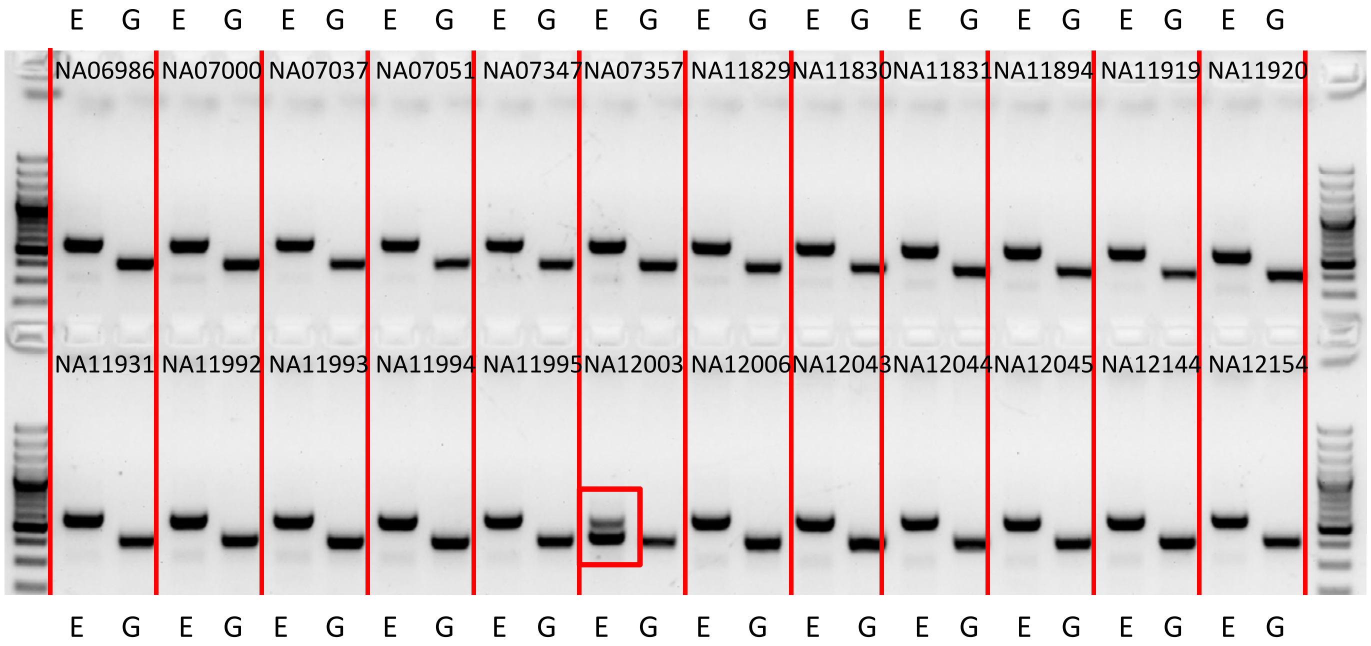

Reference sequence and Sanger sequencing of the 2 products obtained for the E reaction for NA12003 (red box above):
SNP rs8009132 is highlighted (black) and the location of the original 3'-flank primer (SP1Gchr14_52404039, used for the PCR shown on the previous page) overlapping the SNP is shown. The lower band corresponds to the allele without L1 insertion. It carries the G allele of this SNP which suggests that the SNP is responsible for the lower amplificiation efficiency of this allele observed with the original primer (see previous page), eventually resulting in the genotyping error observed for NA120003.

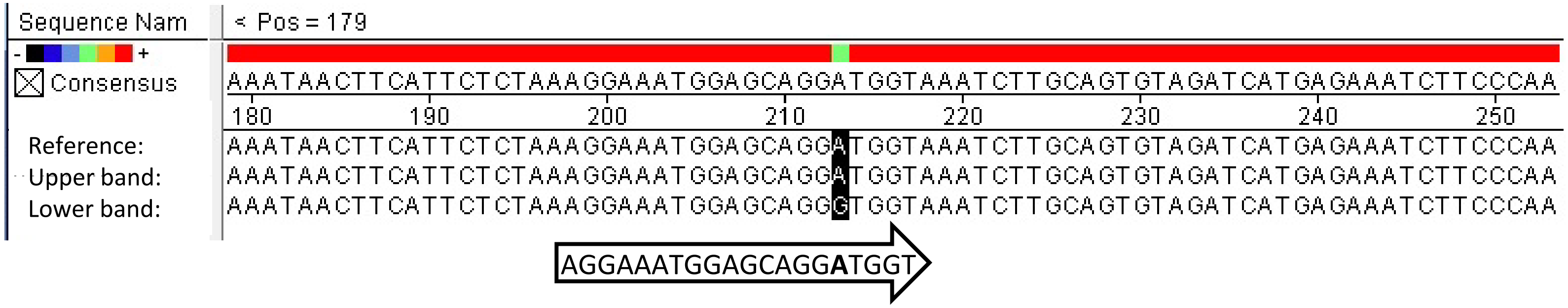

P1_M_061510_18_386

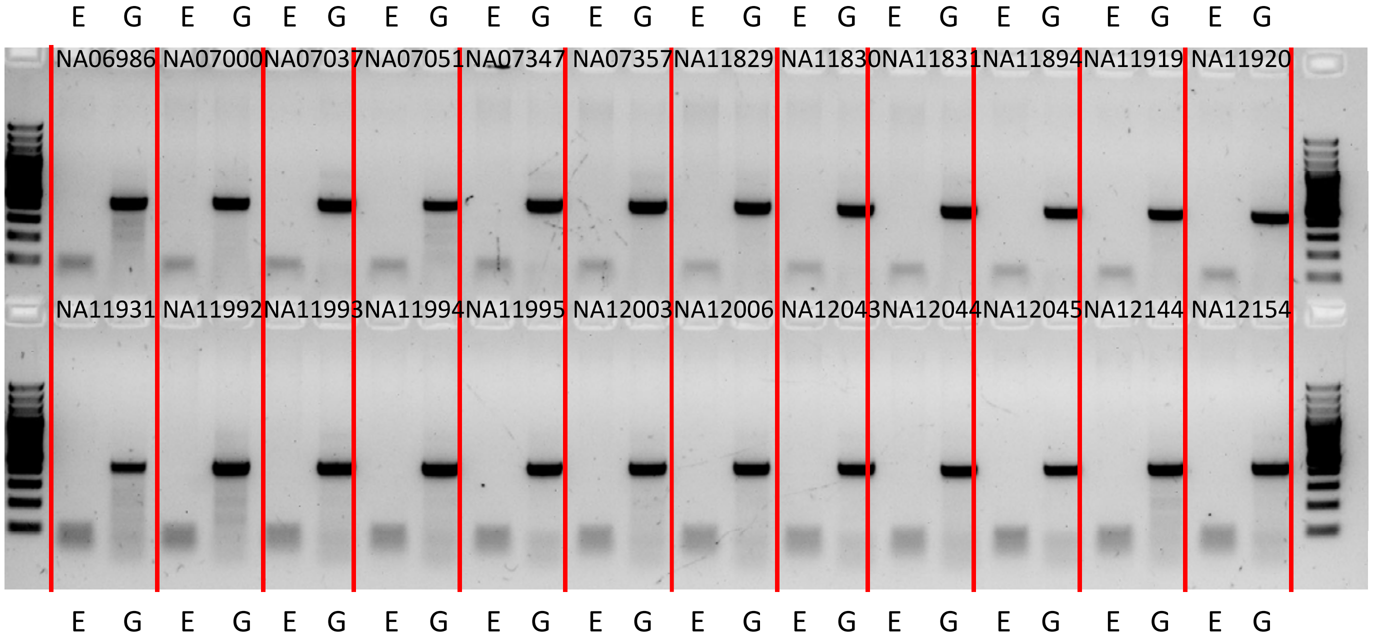

P1_M_061510_18_386

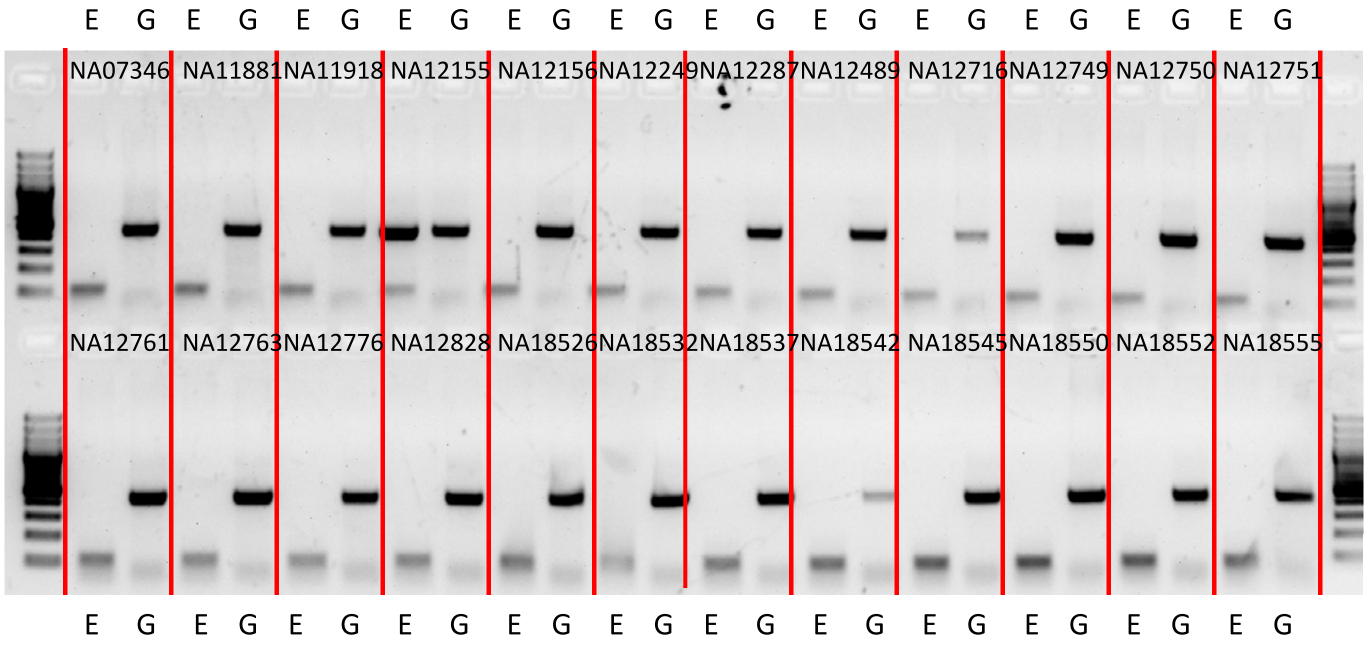

P1_M_061510_20_28

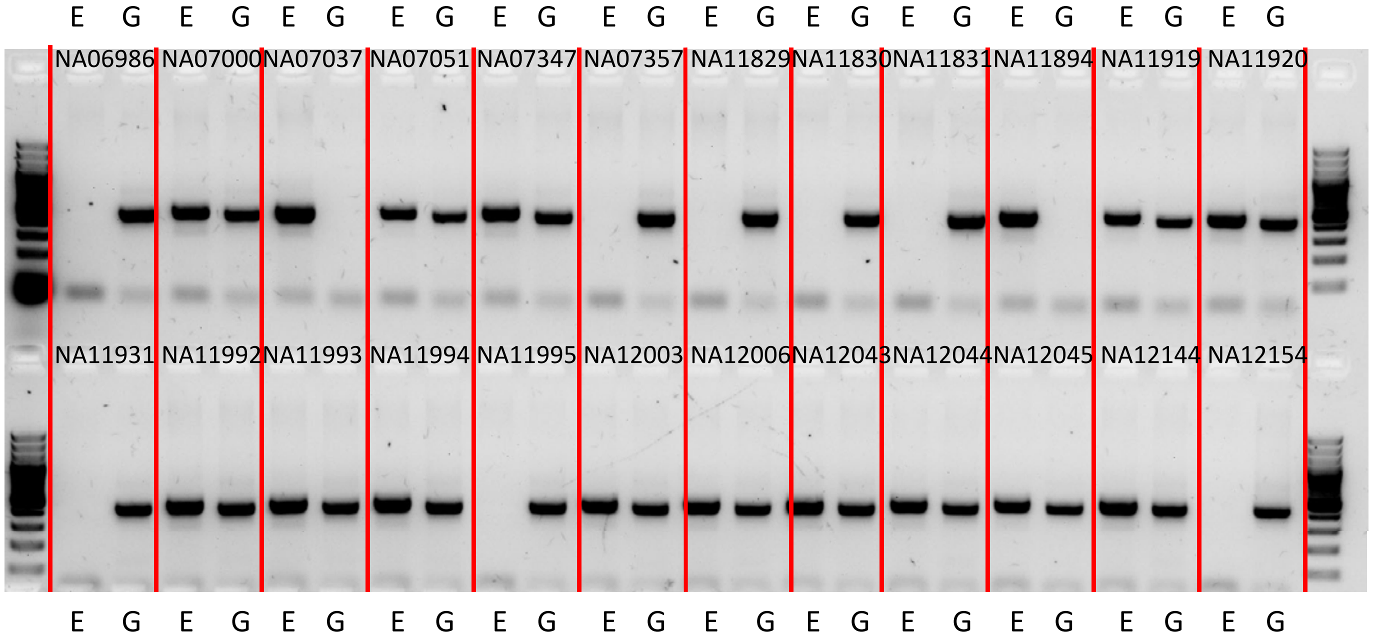

P1_M_061510_20_89

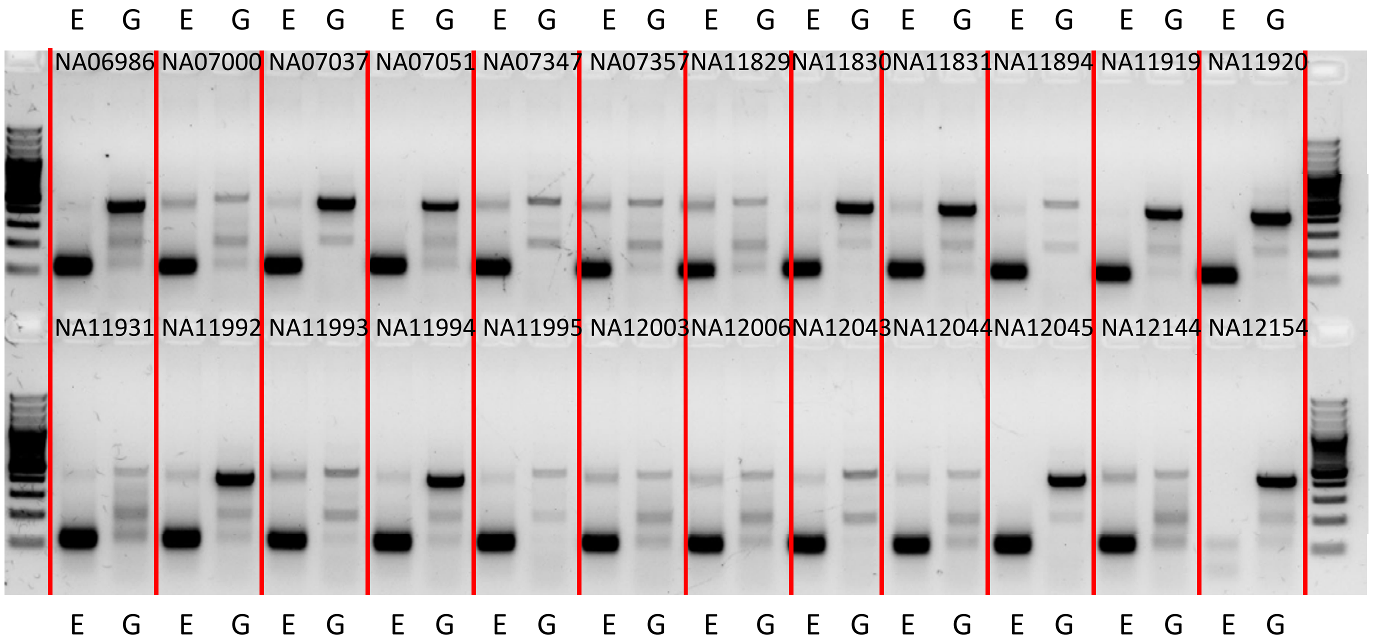

P1_M_061510_20_89

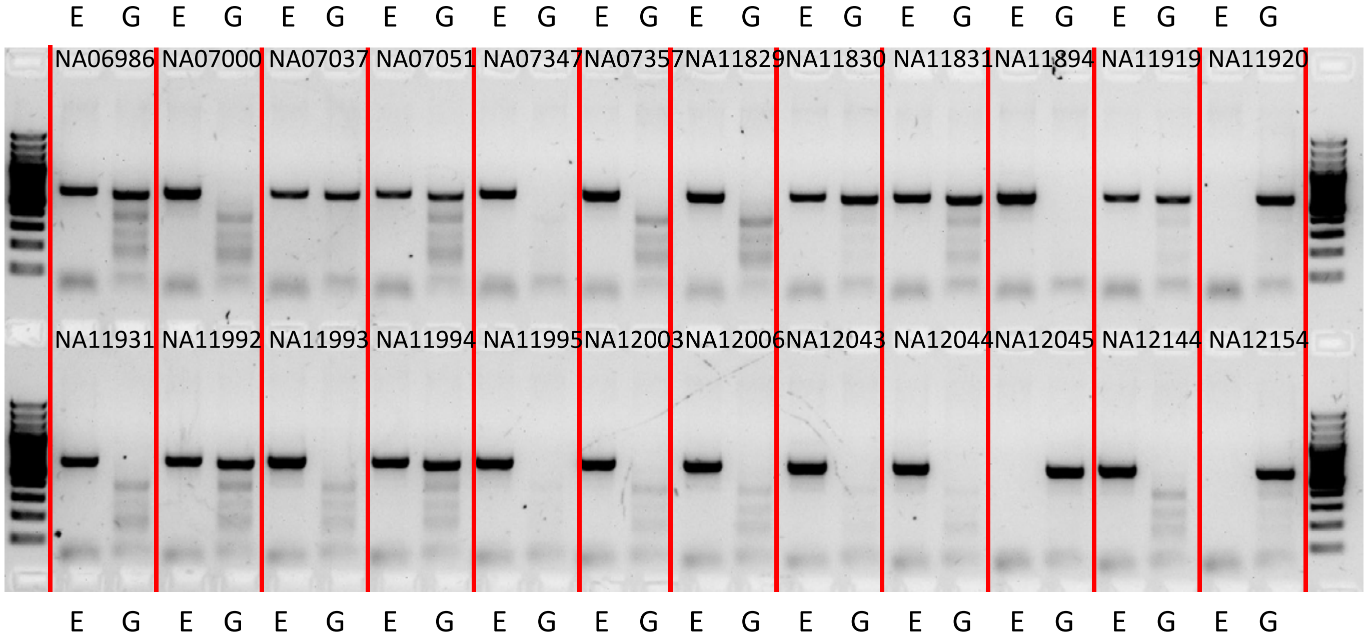

P1_MEI_190&P2_MEI_1442

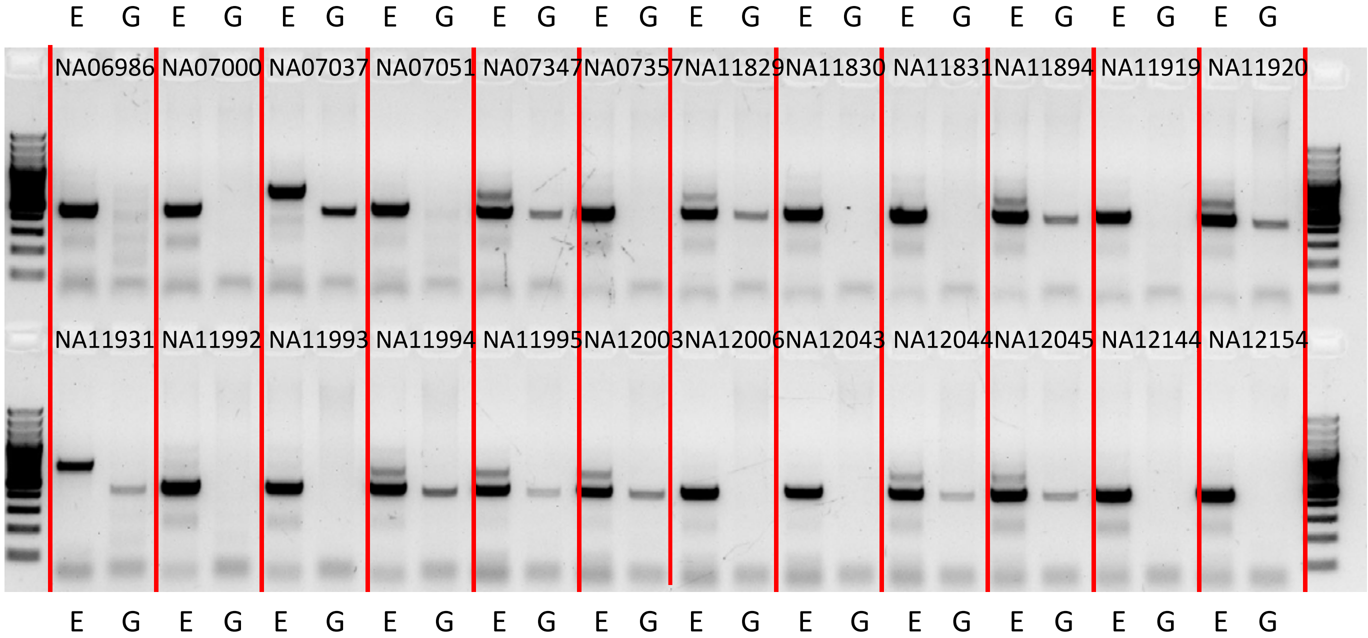

P1_MEI_2893&P2_MEI_1141

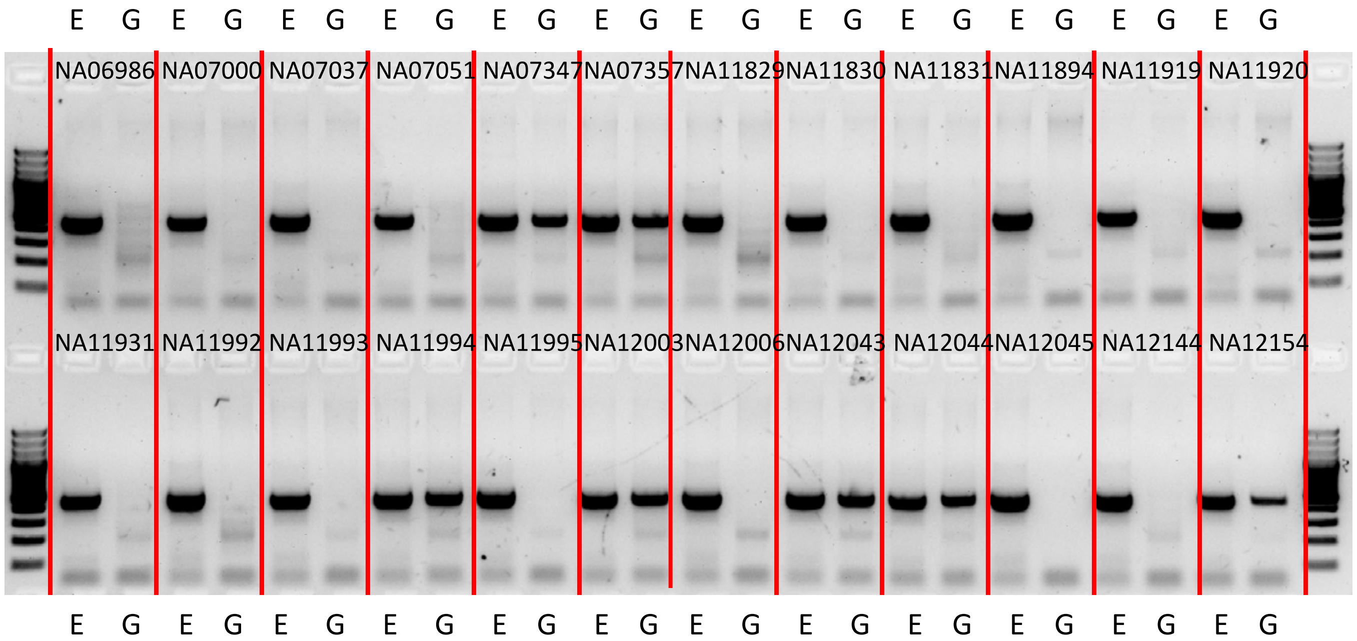

P1_MEI_3120

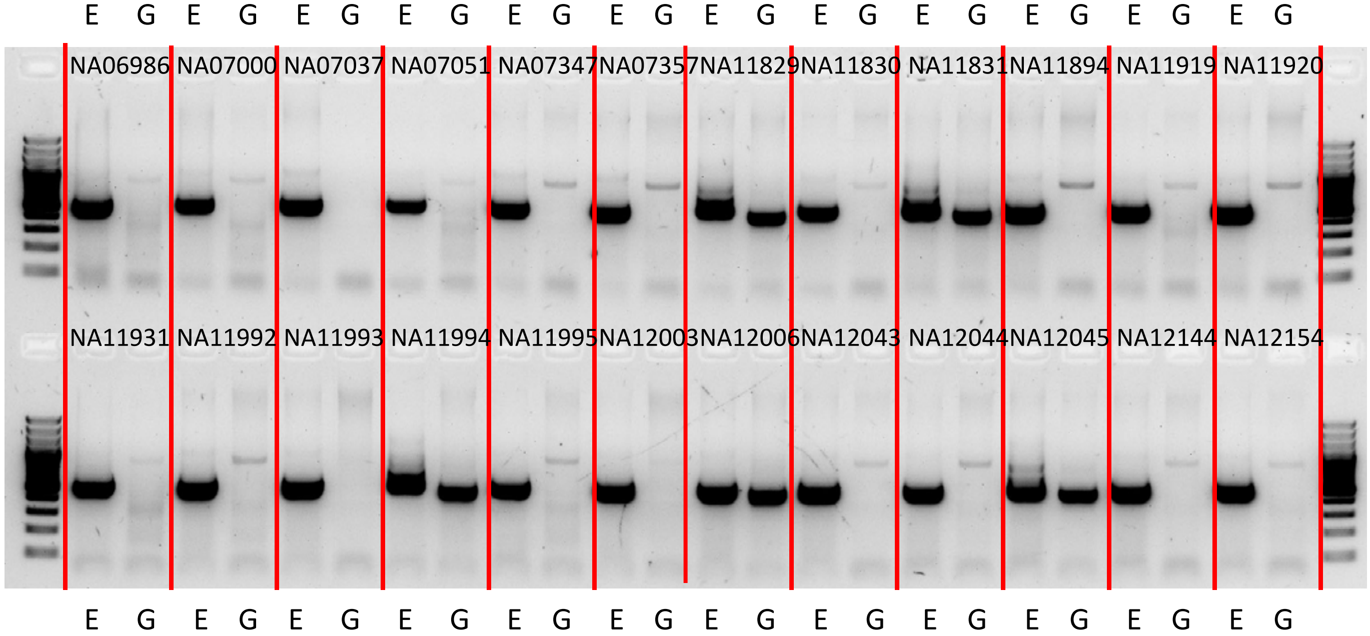
E G E G E G E G E G E G E G E G E G E G E G E G
NA06986 NA07000 NA07037 NA07051 NA07347 NA07357 NA11829 NA11830 NA11831 NA11894 NA11919 NA11920
NA11931 NA11992 NA11993 NA11994 NA11995 NA12003 NA12006 NA12043 NA12044 NA12045 NA12144 NA12154
E G E G E G E G E G E G E G E G E G E G E G E G
