# Supplementary material for: Read count-based method for high-throughput allelic genotyping of transposable elements and structural variants
Source: BMC Genomics. 2015 Jul 8;16(1):508. doi: 10.1186/s12864-015-1700-4 (PMC4494700; doi:10.1186/s12864-015-1700-4)
Supplement: Additional file 12: Figure S8. — Gel electrophoresis of individual PCR reactions (validations) for the Alu genotype calls that were found to be discordant with the calls made by the 1000GP. [file 12864_2015_1700_MOESM12_ESM.pdf]

Gel electrophoresis of individual PCR reactions for Alu genotype calls that were not concordant with the calls obtained from the 1000 Genome Project

The following 68 genotype calls from were discordant in the E libraries and were tested individually (single PCR reactions) and analyzed on agarose gel (page 3-9). The asterisk denotes discordance with the sequencing-based method.

P1_M_061510_1_95 : NA12155, NA12828
P1_M_061510_1_167 : NA11894, NA12751
P1_M_061510_1_400 : NA11894
P1_M_061510_2_124 : NA07037, NA11993
P1_M_061510_2_363 : NA07346, NA07357
P1_M_061510_3_121 : NA07051, NA07357, NA11830, NA11894, NA11993, NA12776
P1_M_061510_3_175 : NA11995
P1_M_061510_3_181 : NA11830, NA12249
P1_M_061510_4_220 : NA11995
P1_M_061510_4_372 : NA12154, NA12828
P1_M_061510_4_430 : NA12144
P1_M_061510_5_173 : NA12155
P1_M_061510_5_271 : NA11992, NA11994, NA12489
P1_M_061510_6_90 : NA12287
P1_M_061510_6_123 : NA11920, NA11992
P1_M_061510_6_149 : NA11881, NA11995
P1_M_061510_6_190 : NA12489
P1_M_061510_6_350 : NA11918, NA12155
P1_M_061510_6_403 : NA12751
P1_M_061510_7_253 : NA12045
P1_M_061510_8_218 : NA06986*, NA07051*, NA07346*, NA07347*, NA07357*, NA11829*, NA11830*, NA11831, NA11918*, NA11992*, NA11995*, NA12003*, NA12154*, NA12156*, NA12249*, NA12716*, NA12751*
P1_M_061510_8_268 : NA11881, NA12155
P1_M_061510_8_305 : NA11919, NA12156, NA12749, NA12828
P1_M_061510_9_111 : NA12144
P1_M_061510_9_132 : NA11994
P1_M_061510_10_57 : NA07346
P1_M_061510_10_286 : NA12156, NA12489, NA12776, NA12828
P1_M_061510_12_68 : NA11894
P1_M_061510_15_100 : NA07346

The following 84 genotype calls from were discordant in the G libraries and were tested individually (single PCR reactions) and analyzed on agarose gel (page 3-9). The asterisk denotes discordance with the sequencing-based method.

P1_M_061510_1_167 : NA12155, NA12750
P1_M_061510_1_228 : NA12716
P1_M_061510_1_334 : NA07347, NA12003
P1_M_061510_1_394 : NA11881
P1_M_061510_1_431 : NA12044
P1_M_061510_2_471 : NA12750
P1_M_061510_3_98 : NA12751
P1_M_061510_3_181 : NA06986, NA11894
P1_M_061510_3_262 : NA12750
P1_M_061510_4_130 : NA12716
P1_M_061510_4_430 : NA11993, NA12249
P1_M_061510_5_145 : NA11831, NA12751, NA12761
P1_M_061510_5_173 : NA07051
P1_M_061510_5_324 : NA11992
P1_M_061510_6_90 : NA11919, NA11993
P1_M_061510_6_350 : NA11830, NA11831, NA11919, NA12003, NA12045
P1_M_061510_7_142 : NA12750*
P1_M_061510_7_253 : NA07037, NA07347
P1_M_061510_9_111 : NA12003
P1_M_061510_9_132 : NA06986*, NA07000*, NA07051*, NA07346*, NA07347*, NA07357*, NA11829*, NA11830*, NA11831*, NA11881*, NA11894*, NA11918*, NA11919*, NA11920*, NA11931*, NA11992*, NA11993*, NA11994*, NA11995*, NA12003*, NA12006*, NA12043*, NA12044*, NA12045*, NA12144*, NA12154*, NA12155*, NA12156*, NA12249*, NA12287*, NA12489*, NA12716*, NA12749*, NA12750*, NA12751*, NA12761*, NA12763*, NA12776*, NA12828*
P1_M_061510_9_158 : NA12154
P1_M_061510_10_128 : NA07346*, NA11881*
P1_M_061510_10_143 : NA11830, NA12716, NA12761
P1_M_061510_11_240 : NA11920, NA12716
P1_M_061510_11_288 : NA12763
P1_M_061510_12_68 : NA12828
P1_M_061510_13_38 : NA11919, NA12750
P1_M_061510_13_177 : NA11830, NA12751

Plate 1

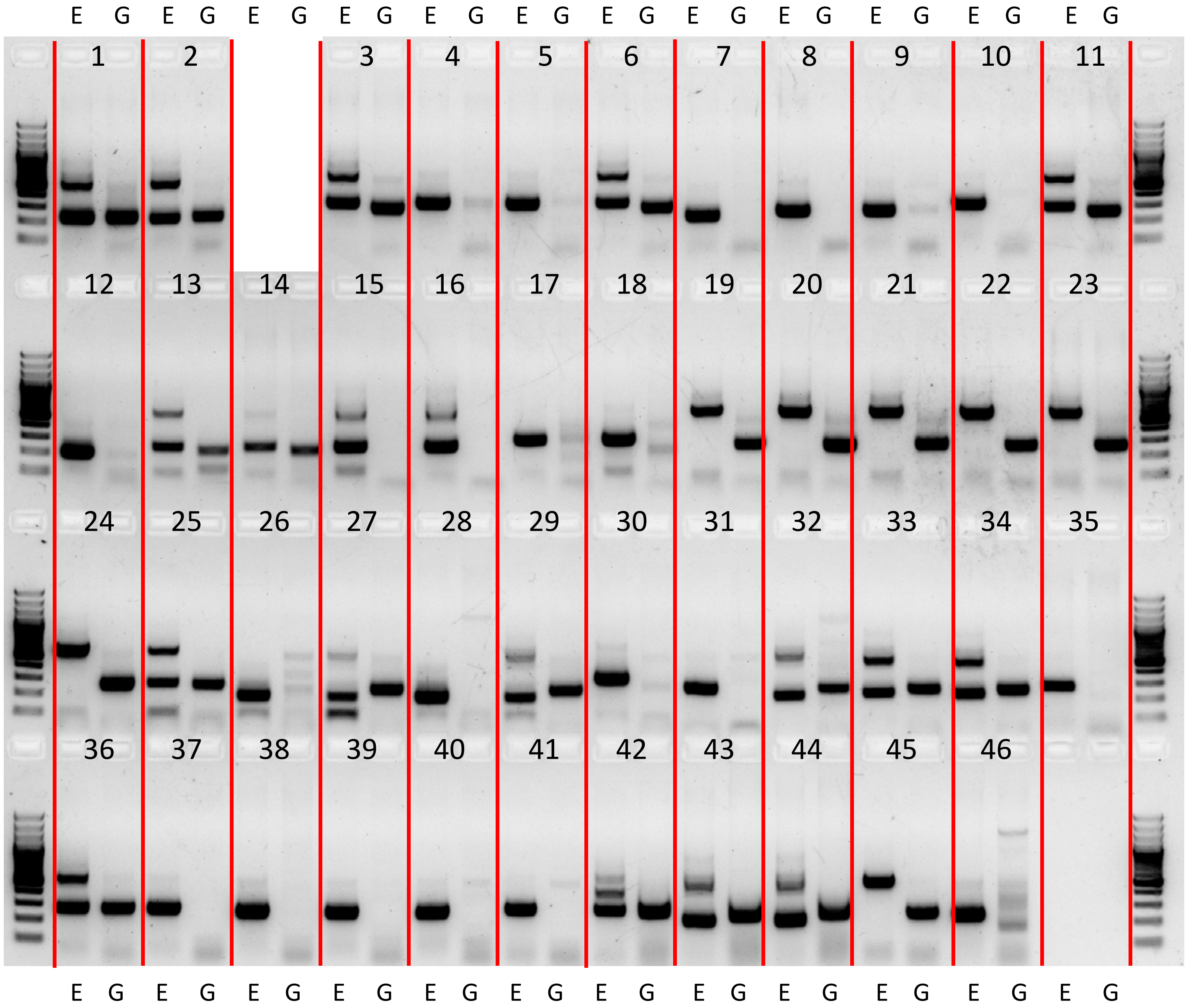
E G E G E G E G E G E G E G E G E G E G E G E G
1 2 3 4 5 6 7 8 9 10 11
12 13 14 15 16 17 18 19 20 21 22 23
24 25 26 27 28 29 30 31 32 33 34 35
36 37 38 39 40 41 42 43 44 45 46
E G E G E G E G E G E G E G E G E G E G E G E G

Lane No.	Locus	Sample
1	P1_M_061510_1_95	NA12155
2		NA12828
3	P1_M_061510_1_167	NA11894
4		NA12155
5		NA12750
6		NA12751
7	P1_M_061510_1_228	NA12716
8		NA07347
9	P1_M_061510_1_334	NA12003
10	P1_M_061510_1_394	NA11881
11	P1_M_061510_1_400	NA11894
12	P1_M_061510_1_431	NA12044
13	P1_M_061510_2_124	NA07037
14		NA11993
15	P1_M_061510_2_363	NA07346
16	P1_M_061510_2_363	NA07357

Lane No.	Locus	Sample
17	P1_M_061510_2_471	NA12750
18	P1_M_061510_3_98	NA12751
19	P1_M_061510_3_121	NA07051
20		NA07357
21		NA11830
22		NA11894
23		NA11993
24		NA12776
25	P1_M_061510_3_175	NA11995
26	P1_M_061510_3_181	NA06986
27		NA11830
28		NA11894
29		NA12249
30	P1_M_061510_3_262	NA12750
31	P1_M_061510_4_130	NA12716

Lane No.	Locus	Sample
32	P1_M_061510_4_220	NA11995
33	P1_M_061510_4_372	NA12154
34		NA12828
35	P1_M_061510_4_430	NA11993
36		NA12144
37		NA12249
38	P1_M_061510_5_145	NA11831
39		NA12751
40		NA12761
41	P1_M_061510_5_173	NA07051
42		NA12155
43	P1_M_061510_5_271	NA11992
44		NA11994
45		NA12489
46	P1_M_061510_5_324	NA11992

Plate 2

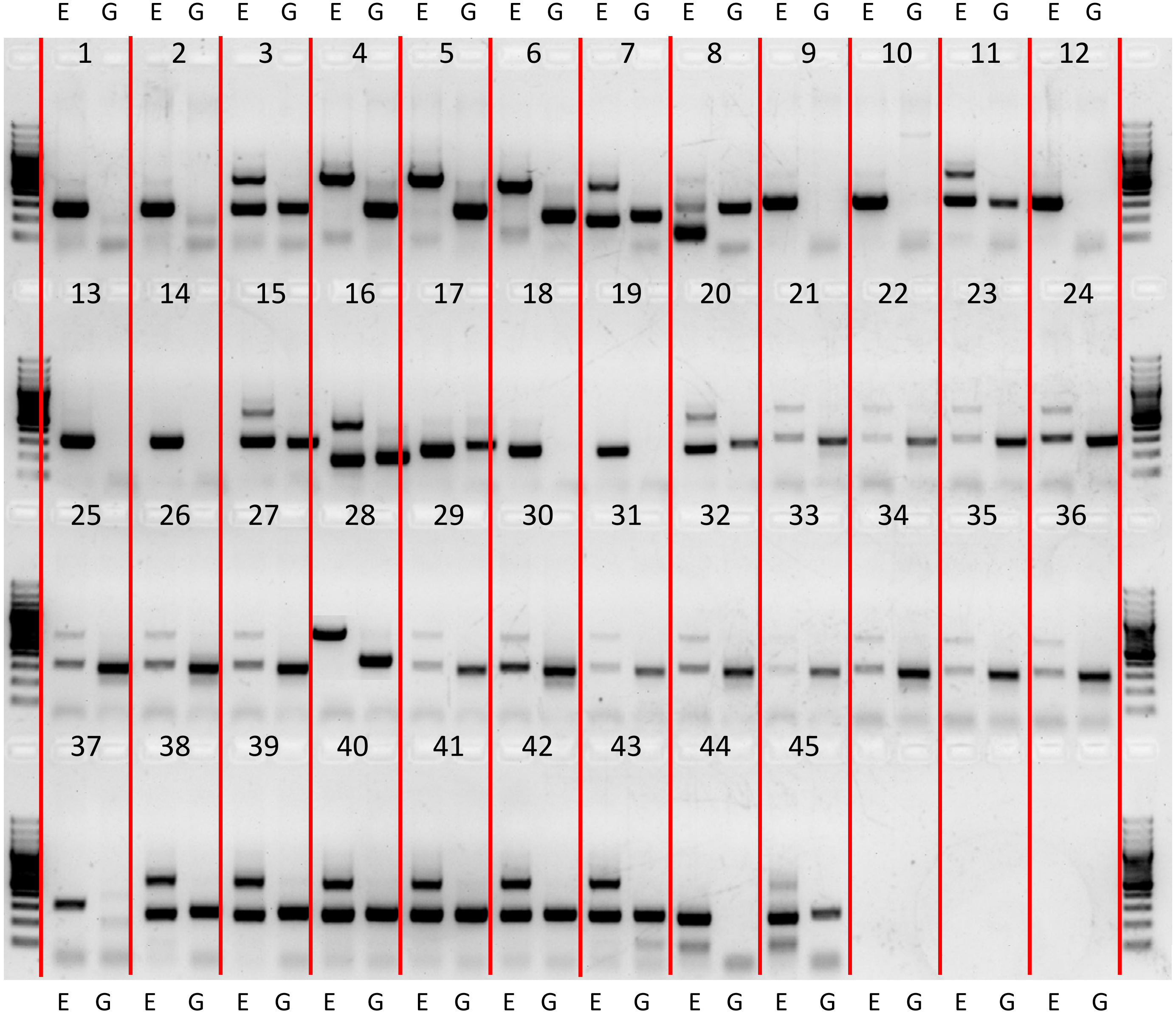
E G E G E G E G E G E G E G E G E G E G E G E G
1 2 3 4 5 6 7 8 9 10 11 12
13 14 15 16 17 18 19 20 21 22 23 24
25 26 27 28 29 30 31 32 33 34 35 36
37 38 39 40 41 42 43 44 45
E G E G E G E G E G E G E G E G E G E G E G E G

Lane No.	Locus	Sample
1	P1_M_061510_6_90	NA11919
2		NA11993
3		NA12287
4	P1_M_061510_6_123	NA11920
5		NA11992
6	P1_M_061510_6_149	NA11881
7		NA11995
8	P1_M_061510_6_190	NA12489
9	P1_M_061510_6_350	NA11830
10		NA11831
11		NA11918
12		NA11919
13		NA12003
14		NA12045
15		NA12155
16	P1_M_061510_6_403	NA12751

Lane No.	Locus	Sample
17	P1_M_061510_7_142	NA12750
18	P1_M_061510_7_253	NA07037
19	P1_M_061510_8_218	NA07347
20		NA12045
21		NA06986
22		NA07051
23		NA07346
24		NA07347
25		NA07357
26		NA11829
27		NA11830
28		NA11831
29		NA11918
30		NA11992
31		NA11995
32		NA12003

Lane No.	Locus	Sample
33	P1_M_061510_8_218	NA12154
34		NA12156
35		NA12249
36		NA12716
37		NA12751
38	P1_M_061510_8_268	NA11881
39		NA12155
40	P1_M_061510_8_305	NA11919
41		NA12156
42		NA12749
43		NA12828
44	P1_M_061510_9_111	NA12003
45		NA12144

Plate 3

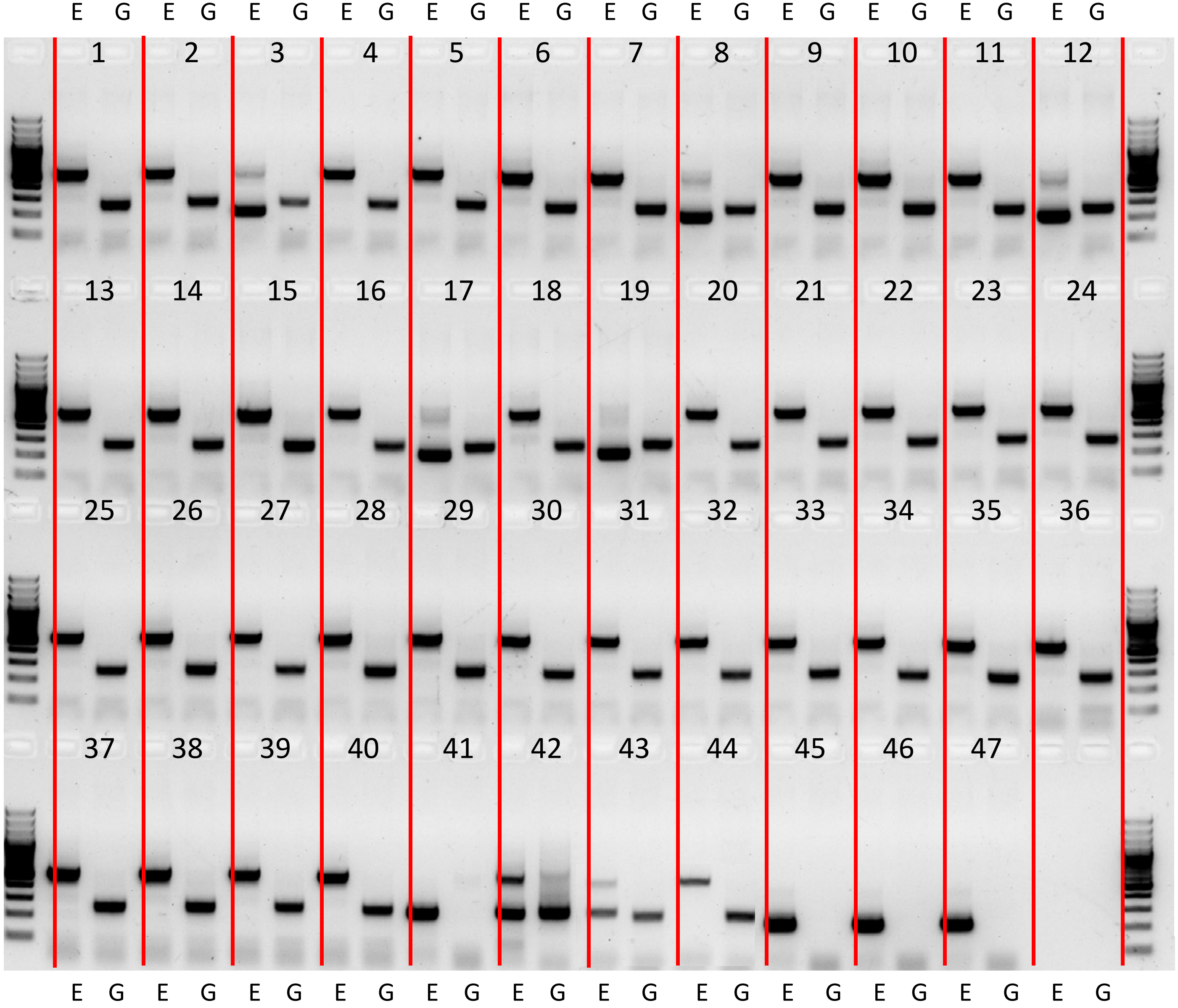

E G E G E G E G E G E G E G E G E G E G E G E G
1
2
3
4
5
6
7
8
9
10
11
12
13
14
15
16
17
18
19
20
21
22
23
24
25
26
27
28
29
30
31
32
33
34
35
36
37
38
39
40
41
42
43
44
45
46
47
E G E G E G E G E G E G E G E G E G E G E G E G

Lane No.	Locus	Sample
1	P1_M_061510_9_132	NA06986
2		NA07000
3		NA07037
4		NA07051
5		NA07346
6		NA07347
7		NA07357
8		NA11829
9		NA11830
10		NA11831
11		NA11881
12		NA11894
13		NA11918
14		NA11919
15		NA11920
16		NA11931

Lane No.	Locus	Sample
17	P1_M_061510_9_132	NA11992
18		NA11993
19		NA11994
20		NA11995
21		NA12003
22		NA12006
23		NA12043
24		NA12044
25		NA12045
26		NA12144
27		NA12154
28		NA12155
29		NA12156
30		NA12249
31		NA12287
32		NA12489

Lane No.	Locus	Sample
33	P1_M_061510_9_132	NA12716
34		NA12749
35		NA12750
36		NA12751
37		NA12761
38		NA12763
39		NA12776
40		NA12828
41	P1_M_061510_9_158	NA12154
42	P1_M_061510_10_57	NA07346
43	P1_M_061510_10_128	NA07346
44		NA11881
45	P1_M_061510_10_143	NA11830
46		NA12716
47		NA12761

Plate 4 and 5

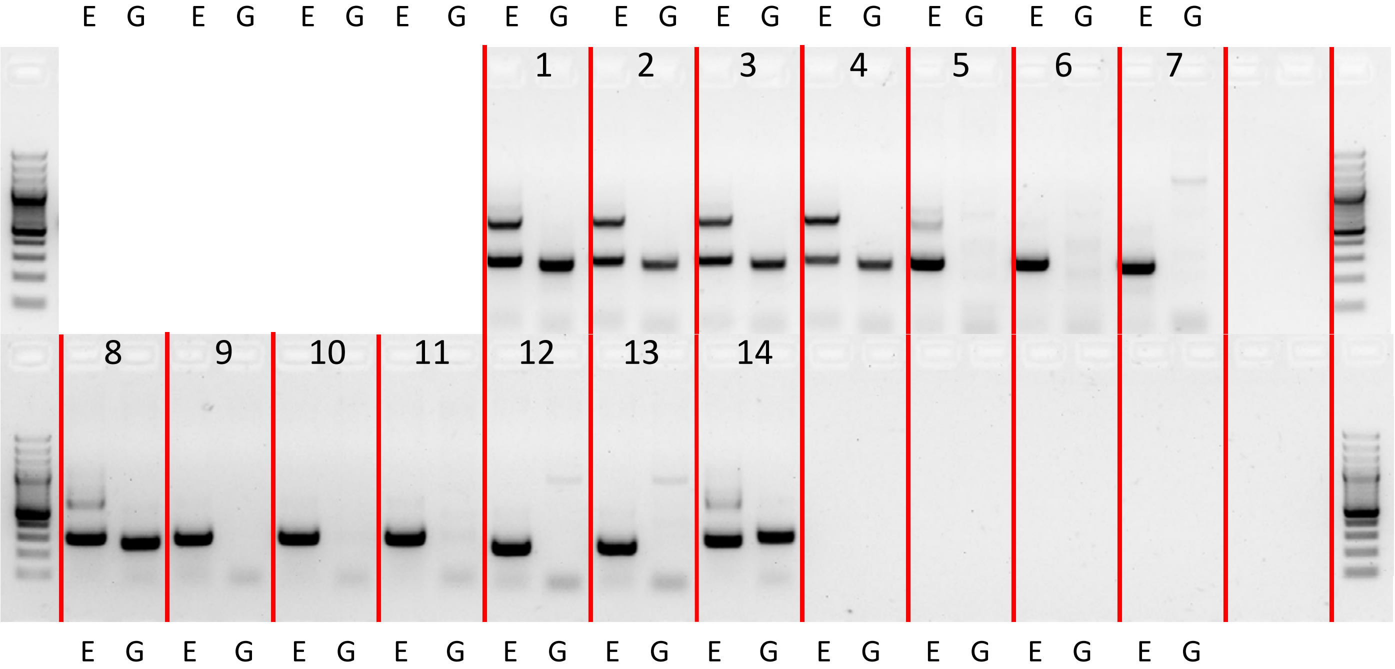

Lane No.	Locus	Sample
1	P1_M_061510_10_286	NA12156
2		NA12489
3		NA12776
4		NA12828
5	P1_M_061510_11_240	NA11920
6		NA12716
7	P1_M_061510_11_288	NA12763

Lane No.	Locus	Sample
8	P1_M_061510_12_68	NA11894
9		NA12828
10	P1_M_061510_13_38	NA11919
11		NA12750
12	P1_M_061510_13_177	NA11830
13		NA12751
14	P1_M_061510_15_100	NA07346
